# ATM suppresses c-Myc overexpression in the mammary epithelium in response to estrogen

**DOI:** 10.1016/j.celrep.2022.111909

**Published:** 2022-12-30

**Authors:** Rifat Ara Najnin, Md Rasel Al Mahmud, Md Maminur Rahman, Shunichi Takeda, Hiroyuki Sasanuma, Hisashi Tanaka, Yasuhiro Murakawa, Naoto Shimizu, Salma Akter, Masatoshi Takagi, Takuro Sunada, Shusuke Akamatsu, Gang He, Junji Itou, Masakazu Toi, Mary Miyaji, Kimiko M. Tsutsui, Scott Keeney, Shintaro Yamada

**Affiliations:** 1 Department of Radiation Genetics, Graduate School of Medicine, Kyoto University, Yoshida Konoe, Kyoto 606-8501, Japan; 2 Shenzhen University School of Medicine, Shenzhen, Guangdong 518060, China; 3 Department of Surgery, Cedars-Sinai Medical Center, Los Angeles, CA 90048, USA; 4 RIKEN Center for Integrative Medical Sciences, Yokohama, Japan; 5 IFOM-the FIRC Institute of Molecular Oncology, Milan, Italy; 6 Department of Medical Systems Genomics, Graduate School of Medicine, Kyoto University, Kyoto, Japan; 7 Institute for Advanced Study of Human Biology (ASHBi), Kyoto University, Kyoto, Japan; 8 Department of Pediatrics and Developmental Biology, Graduate School of Medical and Dental Sciences, Tokyo Medical and Dental University, Bunkyo-ku, Tokyo, Japan; 9 Department of Urology, Graduate School of Medicine, Kyoto University, 54 Shougoin Kawahara-cho, Kyoto 606-8507, Japan; 10 Breast Cancer Unit, Kyoto University Hospital, Graduate School of Medicine, Kyoto University, Kyoto 606-8507, Japan; 11 Graduate School of Medicine, Dentistry and Pharmaceutical Sciences, Okayama University, Okayama, Japan; 12 Molecular Biology Program, Memorial Sloan Kettering Cancer Center, New York, NY, USA; 13 Howard Hughes Medical Institute, Memorial Sloan Kettering Cancer Center, New York, NY, USA; 14 Lead contact

## Abstract

ATM gene mutation carriers are predisposed to estrogen-receptor-positive breast cancer (BC). ATM prevents BC oncogenesis by activating p53 in every cell; however, much remains unknown about tissue-specific oncogenesis after ATM loss. Here, we report that ATM controls the early transcriptional response to estrogens. This response depends on topoisomerase II (TOP2), which generates TOP2-DNA double-strand break (DSB) complexes and rejoins the breaks. When TOP2-mediated ligation fails, ATM facilitates DSB repair. After estrogen exposure, TOP2-dependent DSBs arise at the *c-MYC* enhancer in human BC cells, and their defective repair changes the activation profile of enhancers and induces the overexpression of many genes, including the *c-MYC* oncogene. CRISPR/Cas9 cleavage at the enhancer also causes *c-MYC* overexpression, indicating that this DSB causes *c-MYC* overexpression. Estrogen treatment induced c-Myc protein overexpression in mammary epithelial cells of ATM-deficient mice. In conclusion, ATM suppresses the c-Myc-driven proliferative effects of estrogens, possibly explaining such tissue-specific oncogenesis.

## In brief

Women carrying an ATM gene mutation have an increased risk for estrogen-receptor-positive breast cancer. Najnin et al. highlight the role of ATM in suppressing oncogene overexpression and abnormal cellular proliferation in the mammary epithelium upon estrogen stimuli.

## Graphical Abstract

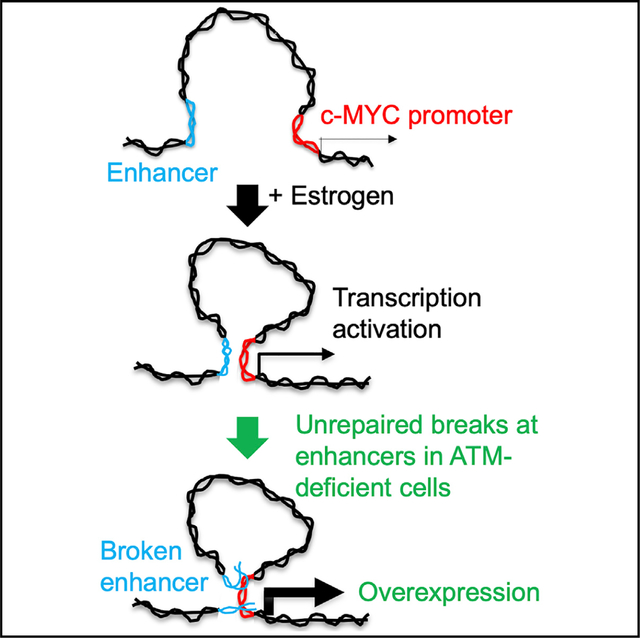

## INTRODUCTION

One in eight women has breast cancer (BC) in their lifetime, and approximately 67%–80% of BCs are estrogen receptor positive (ER^+^).^1,2^ Approximately 5%–10% of BCs are hereditary, with mutations in the *BRCA1*, *BRCA2*, and *ATM* (*ataxia telangiectasia mutated*) genes accounting for most cases.^3–6^ Approximately 0.5%–1% of the general population carries a germline mutation in the *ATM* gene.^7^ Women carrying these germline mutations develop BC with high penetrance after loss of their intact *ATM* allele.^4,5,8,9^ Mechanistically, this loss causes ER^+^ BCs, while loss of the intact *BRCA1* or *BRCA2* allele causes ER-negative BCs.^6,10^ Considering that the loss of heterozygosity (LOH) events occurs at extremely low frequency (approximately 10^−5^ per base) even in cancer cells, the loss of ATM in a small number of cells increases the risk of BC development by five times in females aged younger than 50 years.^11^ The molecular mechanism underlying this dramatic increase in BC development after ATM loss remains unclear.

ATM controls the cellular response to DNA double-strand breaks (DSBs). In response to DSBs, the ATM kinase is immediately activated^12^ to stabilize the p53 tumor suppressor protein via phosphorylation^13^ and stimulate the DNA damage checkpoint pathway.^14,15^ ATM promotes homology-directed repair (HDR), which functions as the dominant DSB repair pathway during DNA replication.^16–19^ Although the damage checkpoint and HDR prevent mutagenesis in all cycling cells, the mechanism by which the phenotype resulting from defective ATM is manifested selectively in estrogen-regulated tissue remains a central unresolved question in ATM biology.

Estrogens bind to ERs α/β and strongly stimulate the proliferation of normal mammary epithelial cells and BCs by inducing *c-MYC* oncogene expression.^20–22^ Because of the extremely high potential for estrogens to stimulate cellular proliferation, ER antagonists are currently the first-line therapy for patients with ER^+^ BC.^23^ Ligand-activated ERs bind to enhancers, leading to the transient transcription of multiple genes.^24–27^ 17β-Estra-diol (E2) exposure causes the recruitment of the FoxA1 transcription factor to genomic DNA independent of ERs. Various growth signals, including estrogens, transiently induce *c-MYC* oncogene expression by controlling clustered enhancer elements known as super-enhancers.^28–30^ c-Myc protein is closely associated with malignant tumor aggression.^31,32^ Even a modest increase in c-Myc expression can significantly enhance oncogenesis in mice.^33,34^

The early transcriptional response to various extracellular signal molecules, including estrogens, cytokines, and neurotransmitters, is achieved by the activation of topoisomerase II (TOP2) catalysis at promoters and possibly enhancers.^35–45^ Of the two known TOP2 enzymes, TOP2A is expressed in cycling cells, while TOP2B is expressed ubiquitously and plays a role in transcription in post-mitotic cells (reviewed in ref.^35^). TOP2 catalyzes strand passage reactions, which involve the movement of one intact double-stranded DNA duplex through a transient enzyme-bridged break in another (gated helix).^28,45–47^ This transient break is also known as a TOP2-DNA cleavage complex (TOP2cc), in which TOP2 is covalently bound to the 5′ end of the DNA DSB, which is repaired by the intrinsic ligation activity of TOP2. The reaction catalyzed by TOP2 occasionally becomes “abortive,” leading to the spontaneous generation of stalled TOP2ccs^48,49^ (reviewed in ref.^45,50,51^). Etoposide, a widely used anti-cancer agent, inhibits this ligation step, generating “stalled” TOP2ccs.^52^

Stalled TOP2ccs are repaired by non-homologous end-joining (NHEJ), the dominant DSB repair pathway in G_0_/G_1_ phase cells.^45^ The direct ligation of stalled TOP2ccs by NHEJ requires prior removal of 5′ TOP2 adducts^53,54^ by tyrosyl DNA phosphodiesterase-2 (TDP2)^55^ and the MRE11 endonuclease^49,53,56,57^ (reviewed in ref.^45,58^). Data suggest that MRE11 collaborates with BRCA1 and CtIP to remove 5′ TOP2 adducts from DSB ends to facilitate NHEJ.^53,59–67^ ATM and TDP2 function independently of each other in the repair of stalled TOP2ccs in primary mouse embryonic fibroblasts at G_1_ phase.^68^ It is unclear whether ATM is required for the repair of stalled TOP2ccs in other cells and tissues when TDP2 is present.

During the early transcriptional response, highly active TOP2 frequently generates spontaneously arising stalled TOP2ccs at promoters.^35–45,69^ The resulting DSBs are ligated via NHEJ.^38,60,70,71^ Whether a similar pattern of stalled TOP2ccs formation occurs at enhancers in this phase is unclear. The effect of these DSBs at enhancers on the transcriptional response when stalled TOP2ccs are left unrepaired is also unknown; investigations of this issue are hindered by difficulties in measuring the activation kinetics of enhancers during the response to extracellular signals.

While genome-wide analyses of epigenetic markers have un-covered many constitutively active enhancers,^72^ the ability to identify enhancers that activate target genes only transiently in response to extracellular stimuli remains a challenge. Nevertheless, the activity of extracellular stimuli-dependent enhancers can be measured by identifying enhancer RNAs (eRNAs), which are bidirectionally transcribed from active enhancers, as their activity correlates with eRNA expression.^73–78^ eRNAs can be detected using an approach known as cap analysis of gene expression (CAGE),^73–78^ in which RNAs are sequenced from the 5′ RNA cap.^79^ However, its sensitivity is limited by the short average half-life of eRNAs (only 1 min), which is approximately 50 times shorter than that of mRNAs.^80^ As a new CAGE method, native elongating transcript-CAGE (NET-CAGE) overcomes this problem by selectively examining nascent RNAs complexed with RNA polymerase II (Pol2) and undergoing productive elongation *in vivo*.^76^ To prevent the release of paused Pol2 during *in vitro* RNA extraction (reviewed in ref.^81^), NET-CAGE involves the extraction of RNA with the Pol2 inhibitor, α-amanitin. These methods allow NET-CAGE to accurately measure the activation kinetics of both individual enhancers and their target promoters in response to extracellular signals.^76^ In the current study, NET-CAGE revealed that the defective repair of stalled TOP2ccs in cells deficient in ATM and TDP2 significantly changed the activation kinetics of enhancers in response to E2. The data led us hypothesize that stalled TOP2ccs occur frequently at estrogen-dependent enhancers of oncogenes and defective DSB repair promotes oncogenesis by causing dysregulation of the response of oncogenes to estrogen.

Here, we aimed to better understand the mechanism by which the risk of oncogenesis is dramatically enhanced upon selective LOH of the *ATM* gene in mammary epithelial cells. To this end, we explored a potential role of ATM in estrogen-mediated oncogenesis in the mammary tissue. In brief, we revealed that ATM is required for repairing stalled TOP2ccs, which occur at enhancers of the *c-MYC* gene in human BC cells during estrogen exposure. Defective repair of such DSBs causes overexpression of the c-Myc protein in human BC cells following E2 exposure and in mammary epithelial cells upon an intraperitoneal (i.p.) injection of E2 into mice, with the high expression continuing for 24 h. Our study highlights the role of ATM in suppressing the oncogenic effect of estrogens and preventing ER^+^ BC development.

## RESULTS

### ATM prevents estrogen-induced genomic instability in mouse mammary epithelial cells

**Figure 1.**
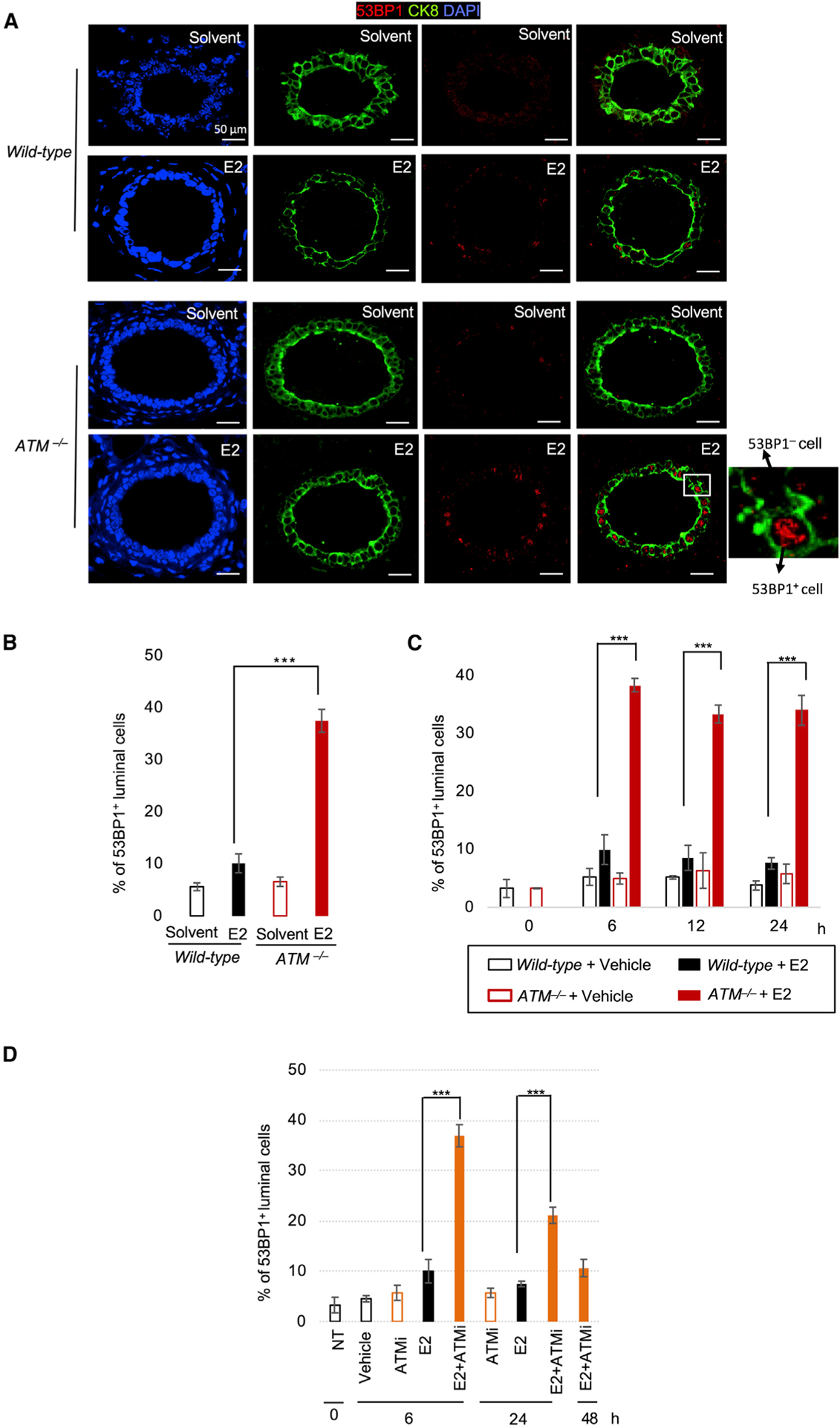
E2 is genotoxic to mammary epithelial cells in *Atm*-deficient mice (A) Cross-section of mammary ducts of female B6 mice carrying the indicated genotypes. Representative images of 53BP1-focus-positive (53BP1^+^) mammary epithelial cells at 6 h after an i.p. injection of E2 or solvent. Cytokeratin-8 (CK8) is a marker of epithelial cells. Scale bar, 50 μm. (B–D) Percentage of 53BP1^+^ epithelial cells at 6 h (B) or indicated hours (C and D) after an i.p. injection of E2 into B6 (B), B6;129 (C), and ATMi-treated B6 mice (D) carrying the indicated genotypes. ATMi was injected together with E2. Examples of histological images for (C) and (D) are shown in Figures S1B and S1C, respectively. Data (B–D) represent mean ± standard deviation from triplicates. ***p < 0.005, unpaired two-tailed *t* test.

We first explored whether ATM promoted the repair of E2-induced DSBs. To this end, we immuno-stained the DSB marker 53BP1 in mouse mammary luminal epithelial cells (Figure 1A). The i.p. injection of E2 (i.p.) into *wild-type* C57BL/6 mice increased the number of 53BP1^+^ epithelial cells by 1.6-fold, while injection of E2 into *Atm*-deficient C57BL/6 mice (hereafter *ATM*^−*/*−^ B6 mice) caused a 6-fold increase (p < 0.005, unpaired two-tailed *t* test) in this population (Figure 1B). Thus, ATM suppresses the genotoxic effect of E2. To exclude the effect of the genetic background (C57BL/6) on this genotoxic effect, we analyzed *ATM*^−*/*−^ mice on a mixed C57BL/6 3 129/Sv genetic background (hereafter *ATM*^−*/*−^ B6;129 mice). Here, we obtained consistent data despite the differential genetic background of each mouse (Figures 1C and S1B). The mammary gland morphology was similar in *wild-type* and *ATM*^−*/*−^ B6;129 mice (Figure S1A). E2-induced DSBs were left unrepaired even at 24 h after E2 injection in approximately 35% of the *ATM*^−*/*−^ epithelial cells (p < 0.005, unpaired two-tailed *t* test) (Figures 1C and S1B), with more than 85% of the cells in the G_0_/G_1_^82^ phases. Collectively, ATM prevents the accumulation of E2-induced DSBs in mouse mammary gland epithelial cells in the G_0_/G_1_ phases.

Compared with the injection of E2 alone, co-injection of the ATM inhibitor (ATMi), KU-55933, and E2 into *wild-type* B6 mice also caused a 5-fold increase (36%–6%)/(10%–4%) (p < 0.005) in the number of 53BP1^+^ epithelial cells detected at 6 h, with more than 50% E2-induced DSBs remaining unrepaired even at 24 h (Figures 1D and S1C). Despite this increase in the number of 53BP1^+^ epithelial cells, there was no increase in the number of ERα-negative non-epithelial cells^22^ (Figure S1D). These data indicate that ATM has a crucial role in repairing E2-induced DSBs in mouse mammary epithelial cells.

### ATM promotes the repair of E2-induced TOP2-dependent DSBs in human BC cells

**Figure 2.**
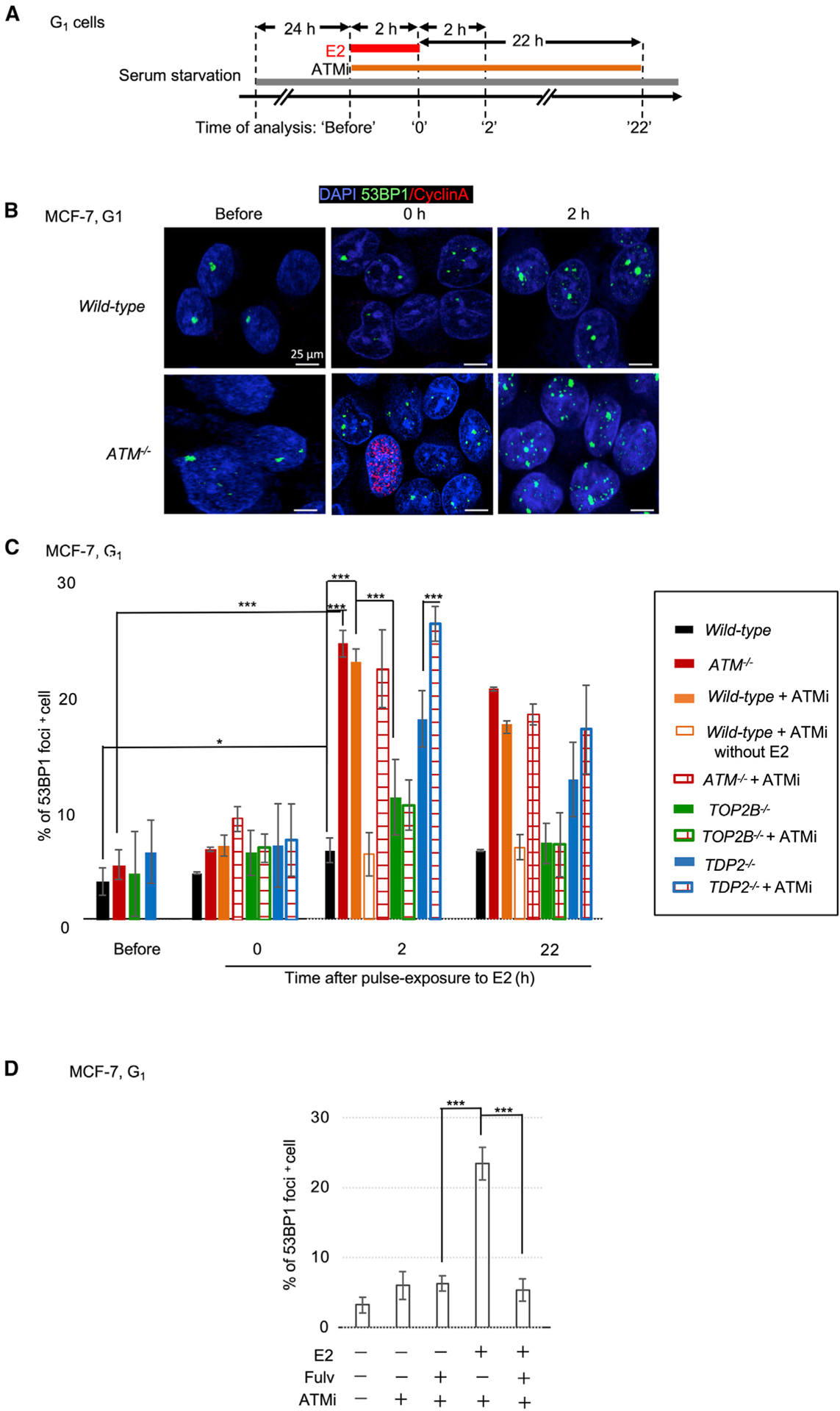
ATM promotes the repair of E2-induced TOP2-dependent DSBs in human BC cells (A) The experimental protocol to analyze E2-induced DSBs in (B) and (C). Serum-starved cells were exposed to E2 and ATMi for 2 h. ‘Before’ indicates time before the exposure, and ‘2 h’ and ‘22 h’ indicate hours for which cells were incubated in E2-free media with or without ATMi after the exposure. (B) Representative image of 53BP1 foci in *wild-type* and *ATM*^−*/*−^ MCF-7 cells at G_1_ phase. Analysis was performed before (left) and after 2 h of E2 exposure (middle) and after 2 h of additional incubation with E2-free media (right). Scale bar, 25 μm. (C) Percentage of G -phase 53BP1^+^ 1 MCF-7 cells (≥10 foci/cell) carrying the indicated genotypes. Data replotted in boxplots are shown in Figure S2C. (D) The inhibitory effect of fulvestrant on E2-induced 53BP1-focus formation in *ATM*^−*/*−^ cells at ‘2 h’ in (A). Data replotted in boxplots is shown in Figure S2G. Data (C and D) are mean ± standard deviation from triplicates. *p < 0.05 and ***p < 0.005, unpaired two-tailed *t* test.

Having determined the prominent genotoxicity of E2 in *Atm*-deficient mice, we next examined whether this finding was relevant to human ER^+^ BC cells and the nature of E2-induced DSBs. To do so, we analyzed E2-induced DSBs in serum-starved MCF-7 human BC cells in the G_0_/G_1_ phases (Figures S2A and S2B; Key Resource Table). We pulsed cells with E2 for 2 h before incubating them in E2-free media for an additional 2 h and 22 h and then counting the 53BP1 foci (Figure 2A). Similar to the *wild-type* and *ATM*^−*/*−^ mouse mammary epithelial cells (Figure 1), only *ATM*^−*/*−^, but not *wild-type* BC cells showed prominent induction of DSBs at 2 h and virtually no resolution of 53BP1 foci, even at 22 h after the removal of E2 (p < 0.005) (Figures 2B, 2C, and S2C). ATMi also increased the number of E2-induced DSBs in *TDP2*^−*/*−^ MCF-7 (Figures 2C, S2C, and S2D) and T47D cells (p < 0.005), another ER^+^ human BC cell line^83^ (Figures S2E and S2F). These data indicate that ATM promotes E2-induced DSB repair in human BC cells and murine mammary epithelial cells in the G_0_/G_1_ phases, independent of TDP2.

We previously showed that E2 induces stalled TOP2ccs in BRCA1-deficient MCF-7 cells.^60,84^ Here, the loss of TOP2B suppressed E2-mediated DSB induction in ATMi-treated cells (p < 0.005) (Figures 2C and S2C). We next investigated whether functional ERs were required for E2-induced DSB formation in *ATM*^−*/*−^ cells. The ER inhibitor, fulvestrant, completely repressed E2-induced DSB formation (p < 0.005) (Figures 2D and S2G). These findings indicate that both TOP2 and functional ERs are required for the E2-dependent DNA damage in *ATM*^−*/*−^ cells in the G_1_ phase, as reported previously in BRCA1-deficient cells.^60,84^ Furthermore, this suggests that stalled TOP2ccs form because of the transcriptional response to activated ERs.

### ATM promotes 5′ TOP2 adduct removal from DSB ends in the G_1_ phase

**Figure 3.**
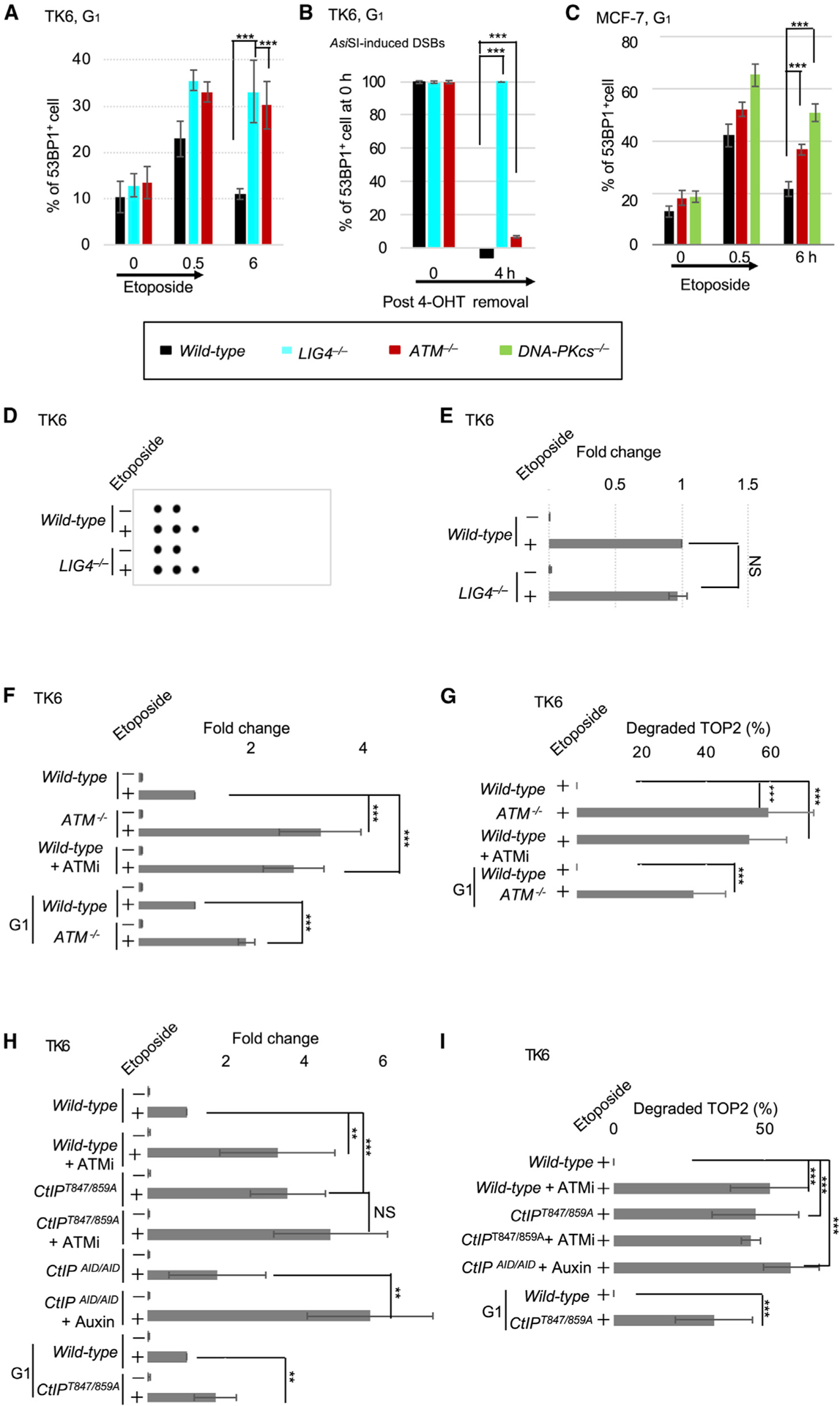
ATM promotes the removal of 5′ TOP2 adducts from DSB ends in the G_1_ phase by phosphorylating CtIP at T847/T859 (A and C) The DSB repair kinetics of G_1_-phase TK6 (A) and MCF-7 (C) cells after a 0.5-h pulse exposure to etoposide. Cells were cultured in etoposide-free medium after the pulse exposure. Percentage of 53BP1^+^ cells was measured (≥5 foci/cell and ≥10 foci/cell for TK6 and MCF-7 cells, respectively). Representative images of TK6 cells are shown in Figure S3C. (B) Proficient repair of ‘clean’ DSBs generated by the *Asi*SI restriction enzyme in *ATM*^−*/*−^ cells but not in NHEJ-deficient *LIG4*^−*/*−^ cells. Cells expressing *Asi*SI fused with ER were treated with 4-OHT for 4 h for DSB induction. The 53BP1 foci were counted in G_1_-phase TK6 cells at 0 h and 4 h after the removal of 4-OHT. The average number of foci in untreated cells was subtracted and subtracted values for 0 h were set as 100%. (D) Representative dot blot analysis of TOP2ccs in TK6 cells of the indicated genotypes treated with etoposide for 2 h. Genomic DNA (50 μg) was separated by cesium chloride gradient ultracentrifugation, and individual fractions were blotted onto polyvinylidene fluoride filters followed by dot blotting using an α-TOP2β antibody (see Figure S3D). The first and the second fractions represent free TOP2. The third fraction contains stalled TOP2ccs having intact TOP2. (E–I) Quantification of TOP2ccs. The whole stalled TOP2ccs (E, F, and H) were quantified for the indicated genotypes. Representative blots are shown in (D), Figures S3E and S3M, respectively. The *x* axis shows the number of etoposide-induced stalled TOP2ccs relative to that in *wild-type*. Stalled TOP2 having degraded TOP2 was quantified in (G) and (I). Representative western blots were shown in Figures S3E and S3M, respectively. The *x* axis shows the percentage of degraded TOP2ccs relative to the whole stalled TOP2ccs in (F) and (H), respectively. Data (A–C and E–I) represent mean ± standard deviation from triplicates. **p < 0.05 and ***p < 0.005. NS, not significant. Student’s *t* test.

We investigated whether ATM significantly contributes to the repair of stalled TOP2ccs in TK6 cells, in which TDP2 is functional.^60,84^ To monitor the DSB repair kinetics in the G_1_ phase, we disrupted the *ATM* gene in TK6 cells (Figures S3A and S3B) and pulse-exposed *ATM*^−*/*−^ and NHEJ-deficient *LIG4*^−*/*−^ cells (Key resources table) to etoposide (Figures 3A and S3C). Both *ATM*^−*/*−^ and *LIG4*^−*/*−^ cells showed similarly severe defects in repairing stalled TOP2ccs (p < 0.05 and p < 0.005) (Figure 3A), indicating a significant contribution of ATM to the stalled TOP2cc repair in the presence of TDP2. To test whether ATM promotes NHEJ and/or the removal of 5′ TOP2 adducts before repair by NHEJ, we next examined the repair of restriction enzyme-induced “clean” ligatable DSBs carrying 3′-OH and 5′-phosphate DSB ends.^53,85^ We expressed a regulatable *Asi*SI enzyme in TK6 cells and transiently activated *Asi*SI to induce DSBs and monitor the repair kinetics in the G1 phase. LIG4, but not ATM, was required to repair *Asi*SI-induced clean DSBs (p < 0.005) (Figure 3B). Thus, the requirement of ATM for repairing stalled TOP2ccs (Figure 3A) suggests that ATM promotes the removal of 5′-TOP2 adducts, but not subsequent NHEJ. *ATM*^−*/*−^ and NHEJ-deficient *DNA-PKcs*^−*/*−^, MCF-7 cells also exhibited a similar delay in repairing etoposide-induced DSBs in the G_1_ phase (p < 0.005) (Figure 3C). Our findings demonstrate that TK6 and MCF-7 cells require ATM for efficient repair of stalled TOP2ccs.

To confirm the ATM-dependent removal of 5′-TOP2 adducts from DSB ends, we measured the amount of stable TOP2ccs in TK6 cells and G_1_-arrested MCF-7 cells. We lysed cells and separated TOP2ccs from free TOP2 in cellular lysates by cesium chlo-ride-gradient ultracentrifugation (Figure S3D). Etoposide-induced stalled TOP2ccs were detected as single or double dots in the third and middle fractions of the TOP2-DNA complex carrying intact TOP2 adducts and TOP2 partially degraded by the proteo-some, respectively (Figures 3D and S3E).^60,84^ *LIG4*^−*/*−^ cells showed no increase in the accumulation of etoposide-induced TOP2ccs (Figures 3D and 3E). Compared with solvent-treated *wild-type* cells, *ATM*^−*/*−^ cells and ATMi-treated *wild-type* cells exhibited 2-fold higher amounts of etoposide-induced TOP2ccs (p < 0.005) (Figures 3F, 3G, and S3E), even when we synchronized the cells in the early G_1_ phase with the CDK4/6 inhibitor, palbociclib (Figure S3F).^86^ The result was reproduced in MCF-7 cells arrested at the G_0_/G_1_ phase (Figures S3G, S3H, and S3I). While *ATM*^−*/*−^ had DSBs carrying partially degraded TOP2, *wild-type* cells did not (Figures 3G and S3I), implying the ATM-dependent removal of TOP2 adducts only after their “debulking” with the proteasome.^87^ Thus, ATM promotes the removal of 5′ TOP2 adducts from DSB ends via a mechanism that is independent of its role in DSB end resection during homologous recombination.

### ATM promotes the removal of 5′ TOP2 adducts by phosphorylating CtIP at T847/T859

ATM promotes DSB end resection of HDR via phosphorylation of CtIP at T847/T859 in the S/G_2_ phases.^88–97^ Although CtIP phosphorylation is hardly detectable in the G_1_ phase,^88^ undetectable or low levels of CtIP phosphorylation can still promote the removal of blocking adducts from DSB ends in a process that is independent of its role in HDR.^59,98–100^ To address the potential role of ATM-dependent phosphorylation of CtIP at T847/T859, we inserted the T847A/T859A point mutations into the *CtIP* allelic genes of TK6 cells to generate *CtIP*^*T847A/T859A*^ cells (Figure S3J). *CtIP*^*T847A/T859A*^ cells were 2-fold more sensitive to etoposide (according to etoposide dose that decreases the percent survival to 10%) (Figure S3K) and exhibited delayed repair of etoposide-induced DSBs in the G_1_ phase compared with *wild-type* cells (Figure S3L). The conditional depletion of CtIP and *CtIP*^*T847A/T859A*^ mutations increased the accumulation of stalled TOP2ccs to the same extent after etoposide exposure (Figures 3H, 3I, and S3M). *CtIP*^*T847A/T859A*^ cells synchronized in the G_1_ phase also showed increased formation of etoposide-induced TOP2ccs (Figures 3H, 3I, and S3M). Collectively, these findings indicate that CtIP promotes the removal of 5′ TOP2 adducts in the G_1_ phase via a mechanism that may require CtIP phosphorylation at T847 and T859.

We next investigated the functional interaction between ATM and the CtIP phosphorylation sites. *ATM*^−*/*−^ and *CtIP*^*T847A/T859A*^ cells showed a similar phenotype in colony survival assays (Figure S3K), and ATMi treatment did not further delay etoposide-induced DSB repair kinetics (Figure S3L) or increase etoposide-induced TOP2ccs formation (Figures 3H, 3I and S3M). These data suggest that ATM promotes etoposide-induced DSB repair by phosphorylating CtIP at T847 and T859. This conclusion was supported by data showing that ectopic expression of the phosphomimetic CtIP mutant transgene, *CtIP*^*T847E/T859E*^, reversed the defective DSB repair in *ATM*^−*/*−^ and *CtIP*^*T847A/T859A*^ cells (Figure S3L). ATM-dependent phosphorylation of CtIP thus promotes 5′ TOP2 adduct removal from stalled TOP2ccs. We propose that this phosphorylation occurs only transiently, is undetectable in the G_1_ phase,^88^ and inhibits excessive resection to facilitate NHEJ.

### Defective repair of stalled TOP2ccs causes dysregulation of estrogen-dependent activation of potential enhancers

We investigated whether the defective repair of stalled TOP2ccs caused dysregulation of E2-induced transcription and eRNA expression. To do so, we leveraged the NET-CAGE method, which quantifies the number of eRNAs and nascent RNAs transcribed from the promoter of protein-coding genes (Figures S4A and S4B).^76^ This method excludes the detection of RNAs derived from the release of stalled Pol2 during RNA extraction from cells (Figure S4A). We first examined the functioning of TDP2 rather than that of ATM, as TDP2 has a specific role in removing 5′ TOP2 adducts from DSB ends.^28,45–47^ Considering the genome instability of MCF-7 cells,^101,102^ we prepared TDP2-positive and -negative cell populations by infecting *TDP2*^−*/*−^ MCF-7 cells (Key resources table) with a viral vector expressing an intact TDP2 or catalytic-dead TDP2 (dTDP2); an empty vector (Mock) was used a control (Figures S4C and S4D). NET-CAGE of the infected cell populations revealed that dTDP2 had only a modest impact on the transcriptional response to E2 (Figure S4E), indicating that the effect of TDP2 expression reflects its catalytic activity in the removal of 5′ TOP2 adducts.

**Figure 4.**
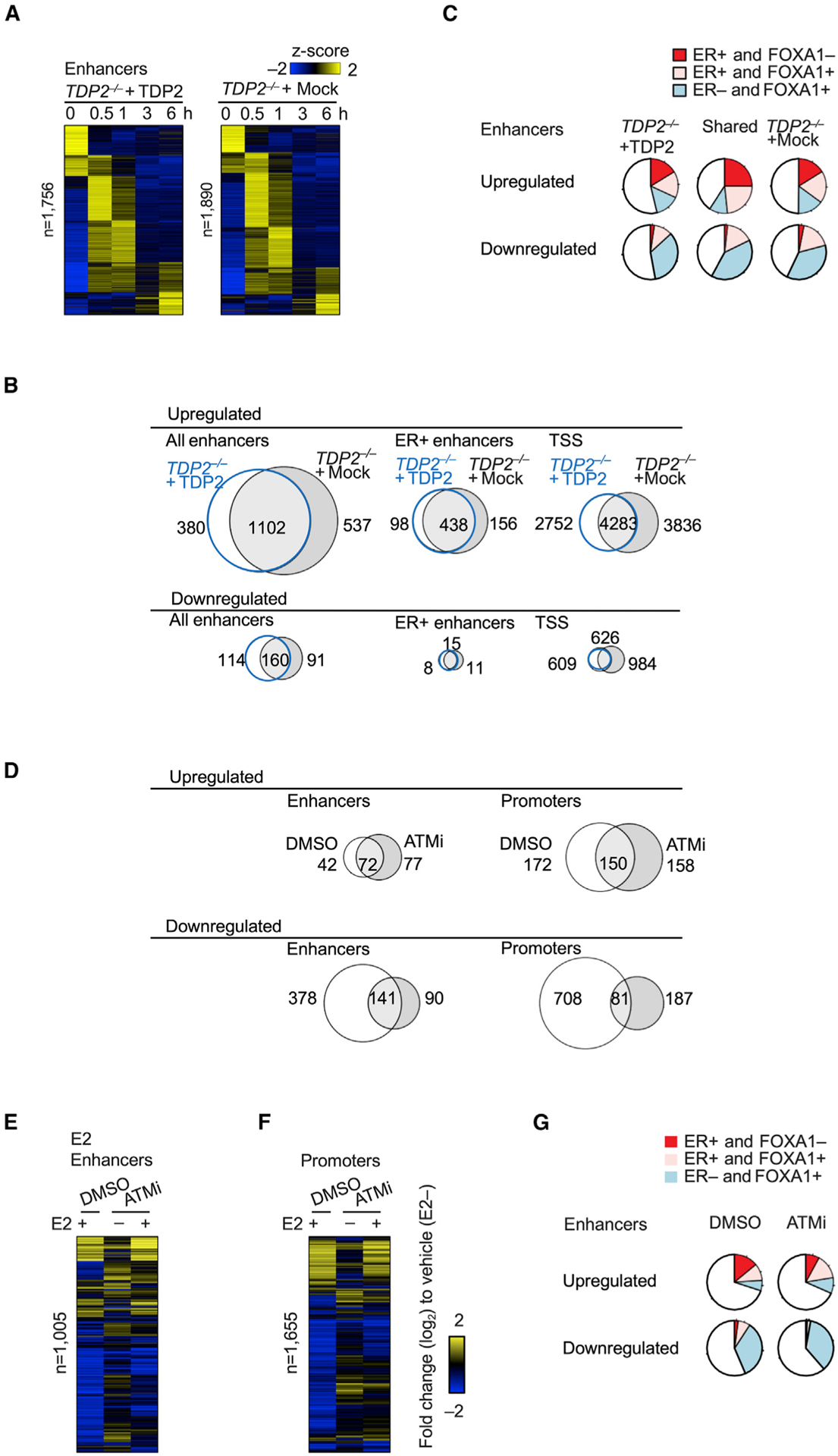
Defective repair of stalled TOP2ccs dysregulates estrogen-dependent activation of potential enhancers (A) Nascent transcriptome analysis at the indicated time-points after the addition of E2. Heatmaps show eRNA expression levels (z scores) in *TDP2*^−*/*−^ cells expressing TDP2 (*TDP2*^−*/*−^ + TDP2, left) or empty vector (*TDP2*^−*/*−^ + Mock, right). Each row represents individual FANTOM5 enhancers that changed the expression of eRNAs with time, defined by TC-seq (p < 0.05). The enhancers were divided into five groups by TC-seq and clustered hierarchically within the groups. See STAR Methods. (B) Area-proportional Venn diagrams showing overlap of upregulated (top) and downregulated (bottom) all enhancers (all enhancers, left), enhancers located within 1 kb of known ERα binding sites (ER^+^ enhancers, middle) and TSSs (TSSs, right). The proportion of ER^+^ enhancers is shown in Figure S4F. (C) Pie charts showing fractions of E2 responsive enhancers localized within 1 kb of the ER and FOXA1-binding sites. (D) Venn diagrams showing the overlap of differentially expressed enhancers (left) and promoters (right) at the 2-h time point between *wild-type* + DMSO and *wild-type* + ATMi. (E and F) Heat maps showing log fold change (log_2_) of enhancer (E) and promoter (F) expression levels at 2 h relative to those at 0 h after exposure of MCF-7 cells to the indicated reagents. (G) Pie charts showing fractions of E2 responsive enhancers associated with the ERα and FOXA1-binding sites for *wild-type* cells treated with DMSO and ATMi.

Triplicate NET-CAGE analyses revealed transient activation of enhancers for only a few hours in response to E2 (Figure 4A). We identified a total of 2,019 eRNAs that were significantly upregulated after E2 exposure in the *TDP2*^−*/*−^*/mock* and *TDP2*^−*/*−^*/TDP2* cell populations (Figure 4B). Most genomic regions identified by eRNAs matched the DNase hypersensitivity site (DHS), H3K27ac, and histone 3 lysine 4 mono-methylation modifications peaks (Figures S4F–S4H). Approximately 50% of these eRNA-expressing sequences were localized within 1 kb of ERα- or FoxA1-binding sites^103^ (Figure 4C). These data support that the transient eRNA expression (Figure 4A) captures genuine E2-responsive enhancers. Expression of the *TDP2* transgene resulted in a time-dependent change in the expression kinetics of eRNAs from these enhancers with ERα-binding sites (Figures 4B and S4I). Similarly, TDP2 expression in *TDP2*^−*/*−^ cells changed the expression kinetics of all the eRNAs (Figures 4B and S4J). Considering the catalytic role of TDP2 in repairing stalled TOP2ccs, these NET-CAGE data suggest TOP2-dependent DSB formation at potential E2-responsive enhancers.

Linking each enhancer to its target gene is a major challenge. To this end, we analyzed the E2-induced activation kinetics of individual transcription start sites (TSSs) of coding genes and eRNAs in our NET-CAGE data. We examined such pairwise expression correlation when TSSs were localized within 400 kb of enhancers, as previously published^75^ (Figure S4K). To interpret the data, we noted that several TSSs are usually present in individual genes, with each TSS usage being differentially controlled by the core promoter immediately upstream of each TSS.^74,76,81,104–108^ The usage of TSSs in each gene is regulated by distinct sets of enhancers. Early transcriptional responses to extracellular stimuli activate enhancers and their target core promoters with similar kinetics; the response of target core promoters is delayed only by 30 min or more. We also found that the usage of 6,843 TSSs changed with kinetics such as the activation kinetics of neighboring (<400 kb) enhancers in the *TDP2*^−*/*−^*/mock* and *TDP2*^−*/*−^*/TDP2* cell populations (Figure S4K), as exemplified by three pairs of potential enhancers and the TSS of neighboring genes (Figures S4L and S4M). The similarity of the activation kinetics between eRNA-expressing sequences and TSSs implies that the former sequences control neighboring TSSs. These data support the formation of stalled TOP2ccs at E2-responsive enhancers leading to alterations in their activation kinetics, which in turn changes the expression kinetics of target promoters.

### ATM loss increases the *c-MYC* transcriptional response to E2 in ER^+^ human BC cells

To explore the role of ATM in the early transcriptional response to E2, we examined the effect of ATMi on eRNA expression at 2 h after E2 exposure. The addition of ATMi changed the E2-dependent expression of eRNAs and coding genes (Figures 4D–4F). Approximately 30% of the E2-responsive eRNAs were derived from sequences near ERα- or FoxA1-binding sites (Figure 4G). These results indicate that stalled TOP2ccs at E2-dependent enhancers arise at E2-responsive enhancers and that their repair is defective in the absence of functional ATM or TDP2, causing dysregulation of eRNA expression during the early transcriptional response to E2.

**Figure 5.**
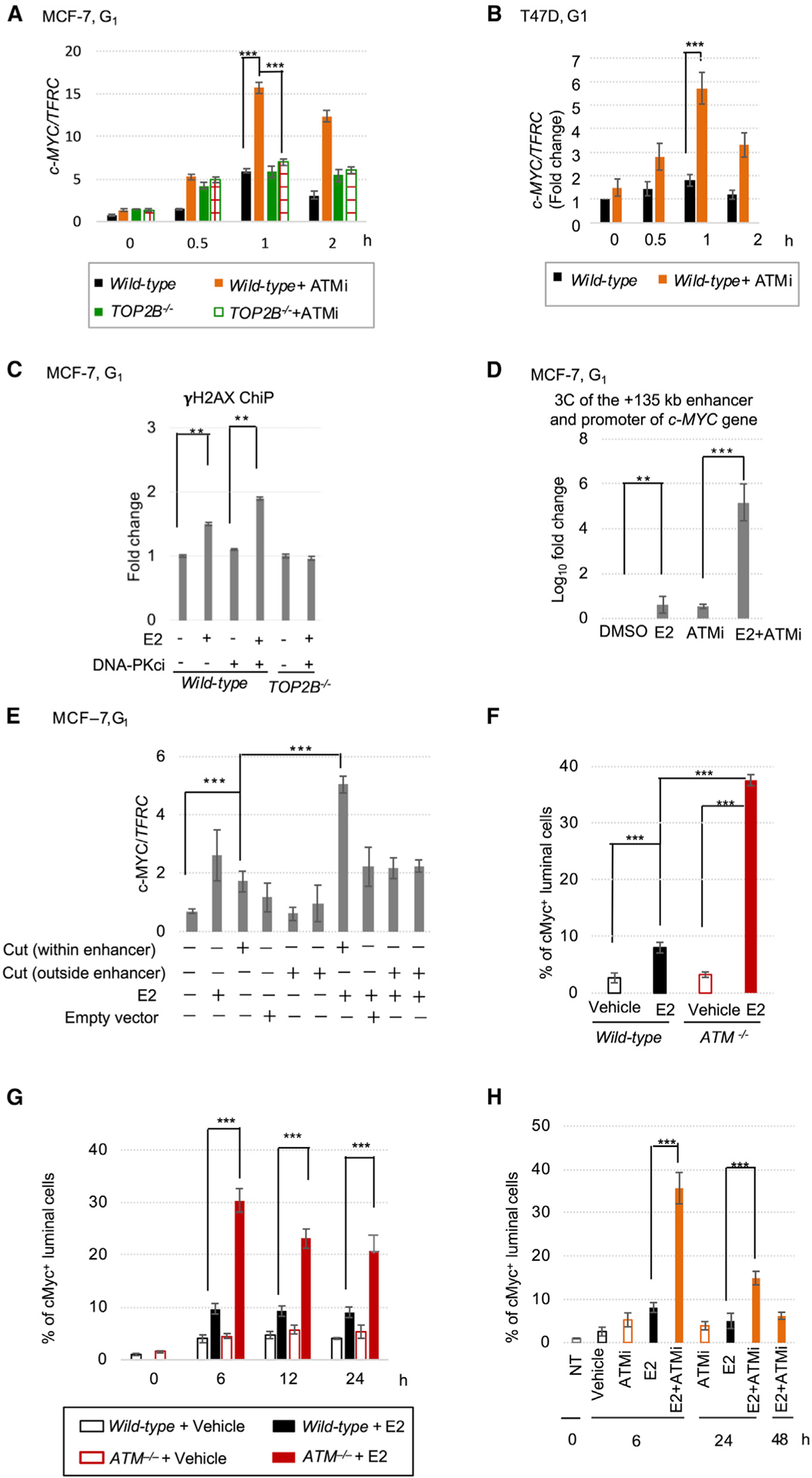
ATM loss increases the *c-MYC* transcriptional response to E2 in ER^+^ human BC and murine mammary epithelial cells (A and B) Kinetics of E2-induced *c-MYC* transcription (normalized to *TFRC*) in G_1_-arrested MCF-7 (A) and T47D (B) cells carrying the indicated genotypes. (C) γH2AX ChiP quantifying E2-induced DSBs in the *c-MYC* E–67 enhancer in G_1_-arrested MCF-7 cells. The *y* axis indicates ChiP fold change over input. (D) 3C analyses of G_1_-arrested MCF-7 cells to measure the extent of interactions between the +135 kb enhancer and the promoter of the *c-MYC* gene under the indicated conditions. The *y* axis indicates fold changes to DMSO-treated data. (E) Quantification of *c-MYC* mRNA (normalized to *TFRC*) after cleavage at the *c-MYC* +135 kb enhancer or two loci outside the enhancer in G_1_-arrested MCF-7 cells. (F–H) Percentage of c-Myc^+^ mammary epithelial cells at 6 h (F) and the indicated hours (G–H) after E2 injection into B6 (F), B6;129 (G), and ATMi-treated B6 (H) mice. ATMi was injected together with E2. Representative images are shown in Figures S5C, S5D and S5F, respectively. Data (A–H) represent mean ± standard deviation from triplicates. ***p < 0.005, **p < 0.05, Student *t* test.

Remarkably, our NET-CAGE data indicated that the addition of ATMi enhanced the E2-dependent expression of *c-MYC* (Figure S4O) and the eRNA expression from the *c-MYC* +135 kb enhancer, which possesses an ERα (ESR1)- binding site, H3K27ac, and DHS^109^ (Figure S4N and S4P). We further examined the E2-induced transcription of *c-MYC* over time in MCF-7 (Figure 5A) and T47D cells (Figure 5B). Inhibiting ATM enhanced the *c-MYC* response to E2, which is consistent with the increase in the *c-MYC* transcriptional response to E2 in the absence of NHEJ reported previously.^84^ Defective repair of stalled TOP2ccs thus causes *c-MYC* overexpression in response to E2.

We also investigated the potential role of TOP2 in the *c-MYC* response to E2. To this end, we examined the *c-MYC* response in *TOP2B*^−*/*−^ cells cultured in the presence or absence of charcoal-stripped serum, the addition of which allows TOP2A expression.^60^ The loss of TOP2B decreased the extent of E2-dependent *c-MYC* induction in ATMi-treated cells (Figure 5A), but not in cells cultured with serum (Figure S5A). Thus, the E2-dependent overexpression of *c-MYC* in the ATM-deficient cells depends on either TOP2A or TOP2B. Collectively, our findings indicate that ATM prevents *c-MYC* overexpression by promoting the rejoining of stalled TOP2ccs arising during the early response to E2.

### Unrepaired DSBs at *c-MYC* enhancer elements cause *c-MYC* overexpression in response to E2 exposure

We hypothesized that stalled TOP2ccs occur at enhancers rather than promoters since local DSBs inhibit the activity of promoters.^110,111^ To test this, we explored DSB formation at a known *c-MYC* enhancer^26^ during E2 exposure by chromatin immunoprecipitation (ChiP). We detected γH2AX ChiP signals after exposing *wild-type* MCF-7 cells to E2 together with DNA-PKi (Figure 5C). γH2AX signals were undetectable in TOP2A-depleted *TOP2B*^−*/*−^ cells and in *wild-type* cells without E2 (Figure 5C). These data support the occurrence of stalled TOP2ccs at the enhancer during the early E2 response. Thus, active eRNA transcription may associate with DSB formation, as early transcriptional response causes DSBs at promoters.^35–45^

To explore the effect of unrepaired DSBs at the *c-MYC* enhancer on the early transcriptional response to E2 in ATM-deficient cells, we performed two experiments at the E2 responsive *c-MYC* +135 kb enhancer (Figure S4N). First, we measured the interaction between the +135 kb enhancer and the *c-MYC* promoter by chromosome conformation capture (3C).^112,113^ The 3C data showed that addition of ATMi increased the E2-induced interaction by approximately 100-fold (p < 0.005) (Figure 5D). Second, we investigated the effect of DNA cleavage by CRISPR/Cas9 on *c-MYC* expression. We infected *wild-type* cells with a lentivirus carrying both CRISPR/Cas9 and guide RNA to cleave the +135 kb enhancer. This cleavage had a synergistic effect with E2 exposure on the induction of *c-MYC* gene expression in MCF-7 (Figure 5E) and T47D (Figure S5B), as previously reported.^114^ Thus, stalled TOP2ccs often form at the *c-MYC* enhancer during the early E2 response. If the breakage is left unrepaired because of an ATM deficiency, it causes *c-MYC* overexpression by facilitating a physical interaction between the enhancer and the *c-MYC* promoter.

### ATM prevents *c-MYC* overexpression in response to E2 in murine mammary epithelial cells

We next investigated whether ATM prevents *c-MYC* overexpression in mouse mammary epithelial cells. After i.p. injection of E2 into *wild-type* and *ATM*^−*/*−^ C57BL/6 mice, we analyzed c-Myc expression in the mammary gland by immunostaining (Figures S5C and 5F). All c-Myc-positive (c-Myc^+^) cells were stained with cytokeratin 8, a biomarker of ERα^+^ luminal epithelial cells^115^ (Figure S5C). E2 injection greatly increased the proportion of c-Myc^+^ cells in *wild-type* and *ATM*^−*/*−^ littermate C57BL/6 mice (3-fold and 11-fold, respectively) (p < 0.005) (Figure 5F). We also injected E2 into *ATM*^−*/*−^ mice bred on a mixed B6; 129 background and confirmed a 3-fold induction of c-Myc^+^ cells in *ATM*^−*/*−^ mice at 6 h after injection compared with *wild-type* (Figures 5G and S5C). Remarkably, *ATM*^−*/*−^ mice showed prolonged c-Myc overexpression even at 24 h (Figures 5G and S5D). This effect was unexpected; the E2 serum concentration returns to background levels at 6 h after injection.^84^ These data indicate that ATM inactivation causes a marked increase in the percentage of c-Myc^+^ cells for 24 h after a single injection of E2.

Co-injection of ATMi with E2 also elevated c-Myc expression even at 24 h but not for 48 h, and the data seemed to be reproducible in the *ATM*^−*/*−^ C57BL/6 mice (Figures 5H, S5E, and S5F). The c-Myc expression kinetics were consistent with the prolonged DSB formation after a single injection of E2 plus ATMi (Figure 1D). In summary, the loss of ATM not only increased the E2-mediated early induction of *c-MYC* gene expression, but also significantly extended its expression in mammary epithelial cells. Thus, ATM is likely required for an appropriate *c-MYC* transcriptional response to estrogens in the mammary gland.

### ATM loss causes abnormal cellular proliferation after daily injection of E2

**Figure 6.**
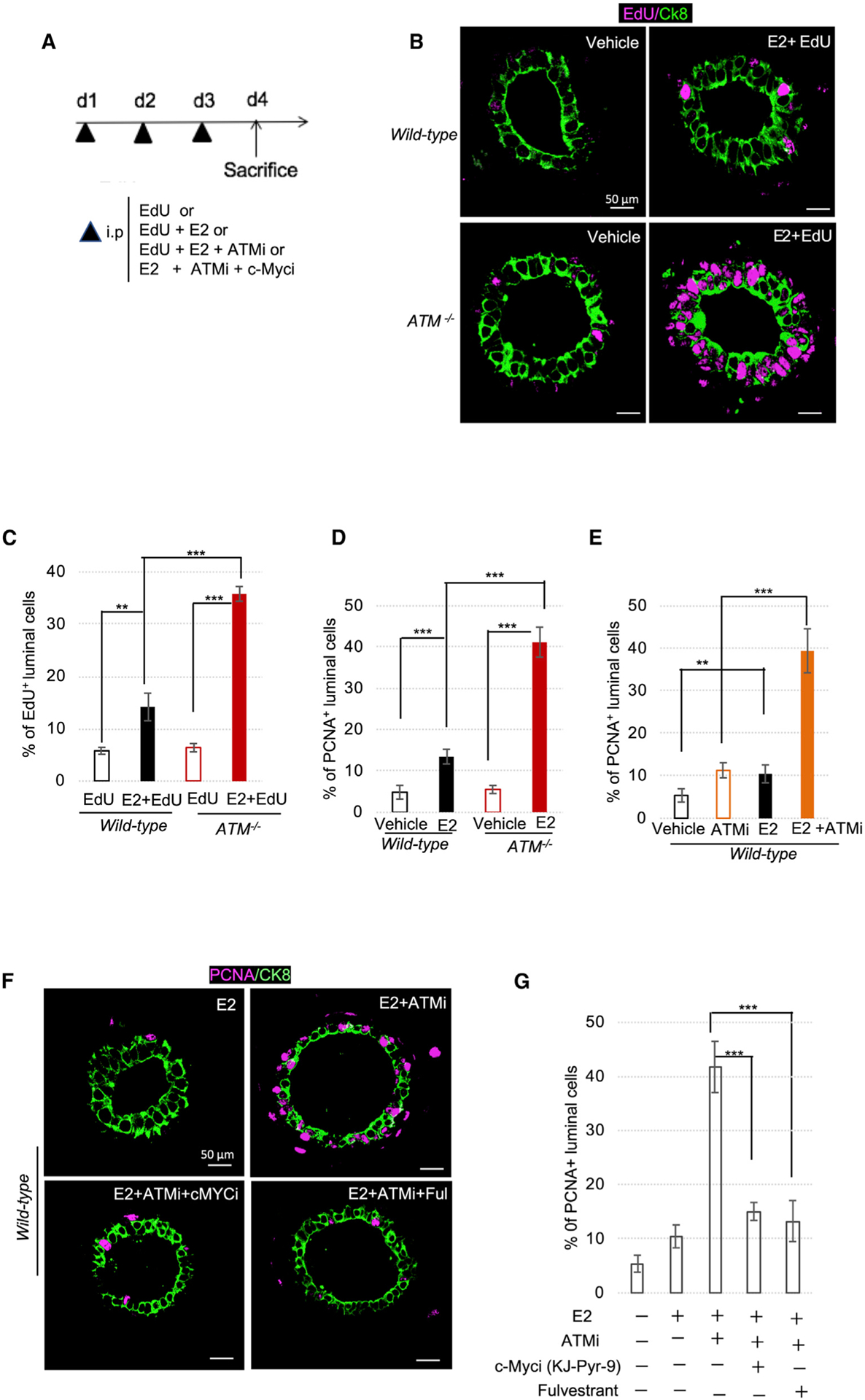
ATM loss causes abnormal proliferation following daily injection of E2 (A) The experimental design for (B–G) and Figures S6A–S6F. The indicated chemicals were i.p. into mice every day for 3 days. Mammary glands were isolated at day 4 to quantify cells proliferating for the last 3 days. (B and C) Representative images (B) and quantification (C) of EdU positive (EdU^+^) mammary epithelial cells (CK8^+^) after injection with the indicated chemicals. (D–G) Percentage of PCNA^+^ mammary epithelial cells (D, E, and G). Representative images are shown in Figures S6E, S6F, and (F), respectively. Data (C–E and G) represent mean ± standard deviation from triplicates. **p < 0.05 and ***p < 0.005, unpaired two-tailed Student *t* test. Scale bar, 50 μm.

In our final assays, we investigated the consequences of an enhanced c-Myc response to E2 in *Atm*-deficient mice injected daily with E2 for 3 days. To identify the cells that proliferated during this period, we co-injected E2 with a nucleoside analog, 5-ethynyl-2′-deoxyuridine (EdU) and examined EdU^+^ mammary epithelial cells on day 4 (Figures 6A and 6B). E2 increased the percentage of c-Myc^+^ cells by 3% and 18% (p < 0.005) by day 4 in *ATM*^*+/+*^ and *ATM*^−*/*−^ mice, respectively (Figures S6A and S6B). E2 also increased the percentage of EdU^+^ epithelial cells by 8% (p < 0.05) and 28% (p < 0.005) in *ATM*^*+/+*^ and *ATM*^−*/*−^ mice, respectively (Figure 6C). Similarly, E2 induced a greater increase (4-fold) (p < 0.005) in the number of PCNA^+^ epithelial cells in *ATM*^−*/*−^ mice compared with *ATM*^*+/+*^ mice (Figures 6D and S6E). The effect of injected ATMi was essentially the same as the loss of ATM in E2-injected mice (Figures 6E and S6F). These data indicate that ATM inactivation causes abnormal mammary epithelial cell proliferation in response to E2.

To test the requirement of c-Myc for this abnormal proliferation (Figures 6C–6E), we co-injected B6 mice with E2 plus ATMi and the Myc inhibitor, KJ-Pyr-9.^116^ Inhibiting c-Myc decreased the number of PCNA^+^ epithelial cells by 2.5-fold (p < 0.005) (Figures 6F and 6G). As expected, co-injection of fulvestrant (an ER inhibitor) completely suppressed the abnormal proliferation induced by E2 and ATMi (Figures 6F and 6G). These findings indicate that ATM prevents the overgrowth of mammary epithelial cells by inhibiting *c-MYC* overexpression in response to estrogens.

## DISCUSSION

The known role of ATM in HDR and the p53 activation at DSBs does not explain a dramatic increase in BC development upon the LOH in women bearing a germline mutation in the *ATM* gene. We, thus, aimed to elucidate why the loss of ATM promotes carcinogenesis specifically in mammary glands. We revealed that ATM inactivation causes *c-MYC* overexpression in E2-treated human BC cells (Figures 5A and 5B). E2 exposure induced DSBs at c-*MYC* enhancers in cells (Figure 5C), and CRISPR/Cas9 cleavage at the enhancers caused *c-MYC* overexpression in DSB-repair-proficient cells (Figure 5E). Our data suggest the frequent occurrence of DSBs at *c-MYC* enhancers during the early transcriptional response to E2 and that a delay in rejoining can cause *c-MYC* overexpression. Like human BC cells, ATM deficiency in mice caused prolonged *c-MYC* overexpression even at 24 h after a single E2 injection (Figures 5G and 5H). This overexpression led to an abnormal proliferative response to estrogens in *Atm*-deficient epithelial cells (Figure 6). We propose that ATM suppresses oncogenesis selectively in mammary epithelial cells by inhibiting c-Myc overexpression in response to estrogens. This newly identified role of ATM, together with the ubiquitous function of ATM and the activation of p53 following the formation of DSBs, explains the high penetrance of ER^+^ BC formation in women carrying germline mutations of the *ATM* gene.^4–6,8,9^

Our study indicated that increased DSBs in ATM-deficient BC cells are caused by the formation of stalled TOP2ccs. We previously demonstrated the formation of stalled TOP2ccs during the early response to E2 and androgen in the mammary and prostate epithelial cells, respectively, of TDP2-deficient mice.^60,117^ In this study, we revealed that the loss of ATM also increased E2-induced DSB formation in murine mammary epithelial cells (Figure 1) and human BC cells (Figure 2). DSB induction depends on TOP2 in ATM-deficient G_0_/G_1_ cells (Figure 2), and ATM promotes the removal of 5′ TOP2 adducts from stalled TOP2ccs, independent of its role in HDR (Figure 3). E2-induced DSBs at *c-MYC* enhancer depended on TOP2B (Figure 5C). Taken together, we conclude that ATM promotes the rejoining of stalled TOP2ccs arising in early E2 response.

Based on previously reported evidence that MRE11 collaborates with CtIP to remove 5′ TOP2 adducts in the G_1_ phase,^59^ we predicted that ATM participates in 5′ TOP2 adduct removal to facilitate NHEJ by phosphorylating CtIP. Here, we show that ATMi increases etoposide-induced TOP2ccs formation in *wild-type*, but not *CtIP*^*T847A/T859A*^, cells in the G_1_ phase (Figures 3H, 3I, and S3M). Ectopic expression of the phosphomimetic CtIP mutant transgene, *CtIP*^*T847E/T859E*^, reversed the defective repair of *ATM*^−*/*−^ and *CtIP*^*T847A/T859A*^ cells (Figure S3L). These data imply that ATM contributes to the removal of 5′ TOP2 adducts by phosphorylating CtIP at DSBs. Nonetheless, the DSB-dependent phosphorylation of CtIP is undetectable in the G_1_ phase.^88^ Conceivably, such limited phosphorylation of CtIP by ATM prevents excessive resection, but still promotes the MRE11-mediated removal of 5′ TOP2 adducts. It has been reported that TOP1 adducts suppress ATM activation at single-ended breakage points^118^; therefore, it can be speculated that the limited phosphorylation of CtIP might result from TOP2-blocking adducts. Collectively, we conclude that ATM promotes the removal of 5′ TOP2 adducts to facilitate NHEJ most likely by phosphorylating CtIP.

Our data suggest the frequent occurrence of stalled TOP2ccs at many E2-responsive enhancers after E2 exposure. Subsequent studies indicated the occurrence of DSBs at enhancers in the absence of TDP2 altered the activation kinetics of 273 enhancers carrying ER-binding sites (Figure 4B). Furthermore, ATM inactivation also changed the E2-dependent induction of eRNAs from 587 potential enhancers (Figure 4D). These data suggest the formation of stalled TOP2ccs during E2 exposure since ATM and TDP2 have a role in repairing stalled TOP2ccs. Our data support the existence of E2-induced DSBs at E2-responsive enhancers of the *c-MYC* gene in *wild-type*, but not in TOP2-deficient, cells (Figure 5C). Considering the requirement of TOP2 in transcription,^28,45–47,119^ the induction of eRNA after exposure to E2 leads to the spontaneous formation of stalled TOP2ccs at enhancers and flanking sequences. Further research is now needed to show the occurrence of DSBs at E2-responsive *c-MYC* enhancers in normal human and murine mammary epithelial cells. A genome-wide analysis of DSB formation during exposure to E2 is also required. Nevertheless, we propose that stalled TOP2ccs form frequently at enhancers in the early transcriptional response to estrogens. The consequence of unrepaired DSBs at enhancers is *c-MYC* overexpression in human BC cells (Figures 5A and 5B) and mouse mammary epithelial cells (Figures 5F–5H).

The role of DSB repair in suppressing the early *c-MYC* response is unexpected, as it is widely believed that unrepaired DSBs suppress transcription from constitutively active promoters near the cleavage site.^110,120–123^ Transcription from DSB ends and the resulting RNA-DNA hybrid formation also causes promoter repression of neighboring genes.^124^ Thus, it is surprising that, although the loss of TDP2 and addition of ATMi increased the occurrence of E2-dependent breakage, this also caused increases in the expression from the promoters of many protein-coding genes (Figures 4B and 4D). The presence of many upregulated genes during the early transcriptional response to E2 implies that most of the TOP2-dependent breakages occur at enhancers, rather than promoters, in this phase. As unrepaired DSBs resulted in both upregulation and downregulation of many genes in DSB-repair-deficient cells (Figures 4B and 4D), we propose that the effect of unrepaired DSBs on the transcriptional response to extracellular signals is far more complex than that on constitutively active promoters near the DSBs.^110,120–123^ Conceivably, unrepaired DSBs at enhancers distantly localized from promoters differentially affect target promoters, depending on the function of enhancers, whether DSBs arise within or outside enhancers, the timing of rejoining, and the distance between broken enhancers and target promoters. While CRISPR/Cas9-mediated cleavage at the E2 responsive enhancer augmented the E2-mediated induction of *c-MYC* expression (Figures 5E and S5B), the effect of such cleavages on signal-induced transcription is likely to vary in individual enhancers and is highly unpredictable. Further genome-wide studies are required to identify hot spots of stalled TOP2ccs and their effects on functional interactions between enhancers and target genes, particularly the *c-MYC* locus.

Women carrying germline mutations of the *ATM* gene are predisposed to ER^+^ BCs when LOH inactivates the *ATM* gene.^4–6,8–10^ LOH events occur at extremely low frequency (approximately 10^−5^ per base) in cells of malignant tumors.^125^ However, once LOH occurs, the resulting ATM loss should dramatically drive selective oncogenesis in the estrogen-regulated tissues. The current study further elucidates the molecular mechanism underlying this selective oncogenesis by revealing the induction of c-Myc overexpression in response to E2 (Figure 5). As a relatively small increase in c-Myc expression significantly enhances oncogenesis,^33,34^ prolonged c-Myc overexpression (Figures 5G and 5H) will effectively drive oncogenesis specifically in mammary epithelial cells. Moreover, known functions of ATM, the activation of p53 by DSBs and the promotion of HDR, explain the enhanced carcinogenesis of ATM-deficient cells. We propose that the combination of *c-MYC* overexpression, a compromised damage checkpoint, and defective HDR is responsible for the extremely efficient and selective oncogenesis in mammary epithelial cells after the loss of functional ATM via LOH.

We proposed that a defect in the repair of breakage at extracellular signal-dependent enhancers as a previously unappreciated mechanism for disease onset based on our demonstration that, in the absence of ATM, defective repair of TOP2-dependent breakage at estrogen-dependent enhancers caused *c-MYC* overexpression (Figures 5C–5E). This mechanism not only explains the increased oncogenesis in mammary epithelial cells, but also the increased risk of metastatic prostate cancers in carriers bearing mutations in the *ATM* genes.^126,127^

We propose that carriers have mutations in the *BRCA1* gene since BRCA1 promotes the removal of 5′ TOP2 adducts before NHEJ.^53,60^ Why carriers having mutations in the *BRCA1* gene develop ER-negative BCs despite the development of ER^+^ BCs upon the loss of ATM remains an important question. The cell origin is a controversial issue, as ER^+^ epithelial cells can undergo de-differentiation to the ER-negative stem-like state during oncogenesis in mice^128–130^ and humans^130^ (reviewed in ref.^131^). A positive feedback loop exists between ER and BRCA1 expression; however, BRCA1 is likely not essential for ER expression.^132^ We propose that ER^+^ BRCA1-deficient epithelial cells are converted to ER-negative BCs through the prolonged ER-dependent DSB formation in BRCA1-deficient ER^+^ epithelial cells during E2 exposure,^60^ which activates ATM and then stabilizes p53, which leads to cell senescence.^133^ Thus, the loss of ER and p53 is likely to confer a considerable growth advantage on BRCA1-deficient malignant cells. In contrast, the loss of ATM weakens the capability of p53 to trigger senescence and renders ER^+^ cells tolerant to estrogen-dependent DSB formation, thereby conferring a growth advantage in response to estrogens. In addition to the enhanced carcinogenesis, unrepaired breakage at extracellular signal-dependent enhancers can cause symptoms of ataxia-telangiectasia (A-T), the autosomal recessive disease caused by *ATM* mutations.^134^ For example, the progressive cerebellar degeneration of people with A-T disorder may result from altered transcriptional responses to neurotransmitters, resulting in the gradual loss of neurons. If dysregulated gene expression causes BC development in carriers bearing *ATM* mutations and contributes to the A-T phenotype, it is possible that ER inhibitors that prevent BC development and gene therapy to correct dysregulated gene expression could relieve the severe symptoms of cerebellar degeneration.

### Limitations of the study

Our study has two major limitations. First, we did not assess tumor formation. Second, we did not show a direct link between the impact of ATM deficiency or resulting *c-MYC* overexpression on BC formation. The overexpression of *c-MYC* alone may not account for the dramatically enhanced BC risk upon LOH of the *ATM* gene in mammary epithelial cells. There may be multiple additional transcriptional changes in ATM-defective mammary epithelial cells upon E2 exposure, due to the persistence of stalled TOP2 lesions on the DNA. Any of these changes could be causal for BC; future studies will need to address all oncogenic changes as well as *c-MYC* overexpression.

## STAR☆METHODS

### RESOURCE AVAILABILITY

#### Lead contact

Further information and requests for resources and reagents should be directed to and will be fulfilled by the lead contact, Shintaro Yamada (yamada@rg.med.kyoto-u.ac.jp).

#### Materials availability

Mutant cells generated in this study are available from the lead contact with a completed Materials Transfer Agreement.

#### Data and code availability

- Data availability: NET-CAGE and other data have been deposited at GEO: GSE218320 and Mendeley Data: https://doi.org/10.17632/v74k5sntdk.1, respectively. The GEO accession number and the link to data at Mendeley Data are listed in the key resources table.
- Code availability: This paper does not report original code.
- Any additional information required to reanalyze the data reported in this paper is available from the lead contact upon request.

### EXPERIMENTAL MODEL AND SUBJECT DETAILS

#### Animal experiments

##### Mice

All animal work was performed in compliance with relevant regulatory standards and was approved by the Animal Research Committee of Kyoto University and the Memorial Sloan Kettering Cancer Center Institutional Animal Care and Use Committee.

Experiments for Figures 1D, S1C, 5H, S5E, S5F, 6E–6G, S6C, S6D and S6F were performed in Kyoto University. *Wild-type* C57BL/6JJmsSlc female mice were purchased from SHIMIZU Laboratory Supplies. The *ATM* mutation^136^ were maintained on the C57BL/6 (B6) background after >15-time backcrosses to B6. All mice were maintained under specific pathogen-free conditions. Eight-to-ten-week old mice were used for experiments.

Experiments for Figures 1A–1C, S1B, 5F, 5G, S5C and S5D were performed in MSKCC. The *ATM* mutation^137^ were maintained on either B6 or a B6 and 129/SV mixed (B6; 129) background. Either 8–10-week old (B6) or 8–13-week old (B6; 129) mice were used for experiments.

##### Cell culture

TK6 (human B cell line) cells were incubated in RPMI 1640 medium supplemented with horse serum (5%), penicillin (100 U/mL), streptomycin (100 μg/mL), and sodium pyruvate (200 μg/mL). MCF-7 (ER^+^ human breast cancer cell line) and T47D (ER^+^ human breast cancer cell line) cells were maintained in Dulbecco’s Modified Eagle Medium (DMEM) containing fetal bovine serum (10%), penicillin (100 U/mL), and streptomycin (100 μg/mL). Lenti-X^™^ 293T cells were maintained in DMEM supplemented with fetal bovine serum (10%), penicillin (100 U/mL), streptomycin (100 μg/mL), sodium pyruvate (200 μg/mL) and L-glutamine. All the cells were maintained at 37°C in a humidified atmosphere under CO_2_ (5%). The cells used in this study is listed in Key resources table.

### METHOD DETAILS

#### Intraperitoneal injection

Intraperitoneal (i.p.) injections of E2 (6 μg), EdU (600 μg), ATMi (KU-55933, 100 μg + 40% Polyethylene glycol 300(PEG 300)), c-Myci (KJ-Pyr-9, 200 μg) and Fulvestrant (100 μg) were performed with 30G needle in the morning.

#### Immunostaining of mammary gland tissue

For the preparation of cryo-sections, the isolated mammary gland was fixed with 4% paraformaldehyde (PFA) in PBS (4°C, 15 min, on a rocking platform). The sample was washed (×3) briefly with PBS and incubated with 30% sucrose in PBS (room temperature, 1–2 h). The sample was embedded in OCT compound and frozen with liquid nitrogen. Cryo-sections (thickness 10 μm) were cut at −50°C and dried prior to fixation with 4% PFA (room temperature, 3 min) and rinsed with PBS.

For the preparation of paraffin sections, the mammary gland was fixed with 4% formaldehyde in PBS (room temperature, 48 h), dehydrated, and embedded in paraffin. Sections (thickness 5 μm) were cut and mounted on slides for either hematoxylin and eosin (HE) staining or immunostaining.

For immunostaining, paraffin sections were deparaffinized, washed with PBS, and rinsed with dH_2_O (distilled water). For heat-induced epitope retrieval, the specimen was put into boiling sodium citrate buffer (10 mM sodium citrate, 0.05% Tween 20, pH 6.0) for (53BP1,CK8, and PCNA staining) and Tris-EDTA buffer (10 mM Tris-HCl, 1.3 mM EDTA, 0.05% Tween 20, pH 9.0) for c-Myc staining), incubated for 40 min and cooled for 20 min.

For the immunostaining of frozen mammary gland section, tissue was first fixed with 4% PFA for 5 min. No deparaffinization and epitope retrieval steps are required for frozen mammary section. Specimens (both paraffin and frozen) were then washed (33) with PBS-T (PBS with 0.05% Tween 20) and blocked with blocking solution (5% goat serum, 4% BSA, and 1% Triton X-100 in PBS) for >1 h (3 h for c-Myc staining) at room temperature. Specimens were incubated with the following primary antibodies in (1:5) blocking solution overnight (15–20 h) at 4°C: α−53BP1antibody (1:200), α-CK8 antibody (1:200), Alexa Fluor 647-conjugated α-PCNA antibody clone PC10 (1:25), and α-c-Myc antibody (1/200). After washing with PBS-T (33), sections were incubated for 1 h at room temperature with the appropriate secondary antibody (Alexa Fluor^™^ 488 goat α-rat for α-CK8 and Alexa Fluor^™^ 594 goat α-rabbit for α−53BP1and α-c-Myc antibody) diluted with blocking solution. Sections were then washed with PBS (32) and counterstained with Hoechst (2 μg/mL in PBS) for 30 min at room temperature. After washing with PBS, the sections were dried and mounted with Fluoro-Keeper containing DAPI (4′, 6-diamidino-2-phenylindole).

#### EdU staining of the mammary gland

Mammary tissue from EdU-injected mice was embedded in paraffin and sectioned (thickness 5 μm). After sections were deparaffinized, washed with PBS, and rinsed with dH_2_O, heat-induced epitope retrieval was performed by placing the specimen into boiling sodium citrate buffer (10 mM sodium citrate, 0.05% Tween 20, pH 6.0) for 40 min followed by cooling for 20 min.

For preparation of the frozen mammary gland section, tissue was then fixed with 4% PFA for 5 min and washed several times with 3% BSA in PBS. No fixation step is required for the paraffin-section. Then tissue was permeabilized with Triton X-100/PBS (0.5%) for 20 min at room temperature. The Click-iT^™^ reaction cocktail (100 mM Tris-HCl, pH 8.5, 1 mM CuSO4, 1 μM Alexa Fluor^®^ 594 Azide, 100 mM Ascorbic Acid) was applied for 30 min while protected from light. After washing in PBS (×2), samples were mounted with Fluoro-Keeper containing DAPI and imaged under a confocal microscope (SP8, Leica Microsystems) with a 40×objective lens.

For double staining (e.g., EdU + CK8), sections were immunostained with α-CK8 antibody (1:200) in (1:5) blocking solution overnight (15–20 h) at 4°C. After washing (32) with 3% BSA in PBS, EdU was detected using the Click-iT reaction cocktail as described above.

#### Quantification of mammary gland’s staining

Immunostained mouse mammary tissue was imaged SP8, Leica Microsystems. 53BP1, c-MYC, PCNA, and EdU positive luminal mammary epithelial cells (CK8^+^) were counted manually. For some specimens with EdU single staining, luminal epithelial cells were defined by their locations in mammary ducts. More than 10 mammary ducts (with approximately 300 mammary epithelial cells) were counted for each experiment. To deal with uneven staining of slides, individual image was taken at their own settings (signal intensities cannot be compared directly between images shown in Figures). The offset function was used to cut off excessive signal. Data were analyzed by unpaired Student’s t-test.

#### CRISPR/Cas9-mediated gene-editing, MCF-7 cell

The gRNAs were inserted into the *Bbs*I site of the pX459 vector for expression under the control of the U6 promoter with co-expression of Cas9 under the chicken β-actin promoter. The sequences of the gRNAs for ATM, TDP2, and TOP2β are shown in (Key Resource Table). For transfection, MCF-7 cells seeded in a 6-cm dish and cultured to approximately 60% confluence and then transfected pX459-gRNA using Fugene HD according to the manufacturer’s protocol. At 24 h post-transfection, puromycin (final concentration 2 μg/mL) was added to the medium and the MCF-7 cells were incubated for a further 48 h. After removing puromycin, the cells were cultured for approximately two weeks to isolate the clones. The gene-disruption events were confirmed by Western blot analysis.

*TDP2*^−*/*−^ cells were generated by targeting exon 2 of the *TDP2* gene. The targeting vector was constructed using pSpCas9(BB)-2A-GFP (PX458), a gift from Feng Zhang (Addgene plasmid #48138).

#### ATM^−/−^ generation, TK6 cell

To generate gene-targeting constructs, we generated left and right arms (approximately 1 kb each) of genomic sequences using the primers listed in Table S1. To generate the left arm, we added the upstream and downstream sequences derived from the *Apa*I site to the 5′ and −3′ ends, respectively, of the PCR-amplified left arm. For this purpose, we added “5′-GCGAATTGGGTACCGGGCC” and “5′-CTGGGCTCGAGGGGGGGCC” to the 5′ end of the upstream and downstream primers, respectively, of the PCR-amplified left arm. To generate the right arm, we added the upstream and downstream sequences from the *Afl*II site to the 5′ and −3′ ends, respectively, of the PCR-amplified right arm. For this purpose, we added “5′-TGGGAAGCTTGTCGACTTAA” and “5′-CACTAGTAG GCGCGCCTTAA” to the 5′ end of the upstream and downstream primers, respectively, of the PCR-amplified right arm. These were then inserted into the *Apa*I and the *Afl*II sites of the DT-ApA/MARKER^R^ vector using GeneArt Seamless Cloning Enzyme Mix according to the manufacturer’s instructions.

The gRNA was inserted into the *Bbs*I site of pX330 vector, which expresses gRNA and Cas9 from the U6 and chicken β-actin promoters, respectively. Exon 40 of the *ATM* gene was targeted for gRNA insertion, as this contains the catalytic site. TK6 cells (8×10^6^) were then co-transfected with the two resulting targeting vectors containing different antibiotic markers (Neomycin #9368) and (Puromycin # 9–369) and pX330-gRNA (CRISPR # 9367) into 8 million. The transfected pX330 expressed the Cas9-gRNA complex, which induced DSBs at the specific locus of the genomic DNA and thus facilitated HR between the genomic locus and the arms of the targeting vectors. Details of the vectors and gRNAs are listed in Key Resource Table.

#### *CtIP*^*T847/859A*^ knock-in (KI) mutant generation

The *CtIP*^*T847/859A*^ point mutant was obtained by generating a knock-in (KI) construct. Two gRNAs (gRNA#1 and gRNA#2) were in serted into the *BbsI* site of the pX330 expression vector. gRNA#1 and #2 (Table S1 for sequence information) were designed in introns 17 and 19, respectively. The left arm (1,743 bp) started at 1,134 bp upstream of the gRNA#1 cutting site (but excluded the gRNA sequence) and included exons 18 and 19. The point mutation sites CtIP^T847A^ and CtIP^T859A^ were included in exon 18 to locate the mutations in the left arm. The right arm (1,158 bp) started downstream of the gRNA#2 cutting site (Figure S3H). The mutation-containing cDNA vectors (# pTP2630) were a gift from Dr. Tanya Paull.

The left arm, marker, and right arm were then inserted into the expression plasmid to generate two targeting vectors (Neomycin and Puromycin containing).

For expression in human TK6 cells, the targeting vectors were stably inserted using the Neon (MPK5000) transfection method (pulse voltage: 1500 V; pulse width: 20 ms; pulse number: single).

For expression in KI cells, the cells were first selected using both of the selection markers (neomycin and puromycin) and then checked for the correct insertion of the left and right arms by PCR amplification from the genomic DNA of resistant colonies. Insertion of both the mutations in the cDNA of the transfected cells was then confirmed by sequencing. Details of all the primers are shown in Table S1.

#### CtIP^T847/859E^ and TDP2 overexpression

CtIP^T847/859E^ cDNA was overexpressed in *ATM*^−*/*−^ and *CtIP*^*T847/859A*^ cells to generate *ATM*^−*/*−^*/CtIP*^*T847/859A*^ and *CtIP*^*T847/859A*^*/CtIP*^*T847/859E*^. *ATM*^−*/*−^ *and CtIP*^*T847/859A*^ cells were transfected with cDNA containing the CtIP^T847/859E^ mutation (# pTP3890, a gift from Dr. Tanya Paull) by lentivirus-mediated infection. 24 h after the virus infection, the infected cells were then enriched by puromycin (0.5 μg/mL) selection as the plasmid contains puromycin. The lentiCRISPRv2-puro vector was used to overexpress TDP2 and dTDP2 (cDNAs^55^ were gifts from Dr. Felipe Cortés-Ledes).

#### E2, etoposide, ATMi and DNA-PKi treatment

MCF-7 and T47D cells were first cultured for 48 h in phenol-red-free DMEM containing 10% FBS. For immunostaining, MCF-7 cells were synchronized in the G_1_ phase pre-incubation in a serum-free medium for 24 h before treatment with E2 (10 nM), ATMi (KU55933; 10 μM) and DNA-PKi (NU7441; 10 μM) or etoposide (10 μM). Details of all the reagents are listed in Key resources table.

#### Immunostaining

MCF-7 and T47D cells synchronized in the G_1_ phase were treated with DNA damaging agents and inhibitors (E2, etoposide, ATMi, DNA-PKi). For immunostaining, cells were fixed with methanol for 20 min and permeabilized with Triton X-100 (0.5%) in PBS. After incubation in blocking solution (5%, BSA in PBS), cells were incubated overnight at 4°C with the following primary antibodies: α−53BP1 (1:1,000) and α-Cyclin A (1:500). After washing several times with PBS, cells were incubated with the appropriate secondary antibodies (1:1,000) for 1 h at room temperature. After washing several times with PBS, the section was dried and mounted in Fluoro-Keeper containing DAPI.

TK6 cells were treated with 10 nM etoposide for 30 min, washed (32) with warm PBS and then cultured in drug-free media. For immunostaining, cells were collected using Cytospin and fixed with formaldehyde (4%) in PBS followed by permeabilization with Tween 20 (0.1%) in PBS. After incubation in blocking solution (5%, BSA in PBS), cells were incubated overnight at 4°C with the following primary antibodies: α−53BP1(1:1,000) and α-Cyclin A (1:1,000). After washing several times with PBS, cells were incubated with the appropriate secondary antibodies (1:1,000) for 1 h at room temperature. After washing several times with PBS, the section was dried and mounted in DAPI.

#### ER-*Asi*SI overexpression in TK6 cells

To obtain TK6 cells stably expressing regulatable *Asi*SI ER coupled to an estrogen receptor (ER-*Asi*SI) in cells,^53^ the lentiviral lenti-CRISPRv2 vector containing both ER-*Asi*SI and puromycin-resistance genes (a gift from Gaëlle Legube and Tanya Paull) was transfected into the LentiX-293T cells. The lentiviral particles were harvested at 48 h post-transfection and used to infect into the TK6 cells. To induce DSBs, cells expressing *Asi*SI fused with ER were treated with 4-OHT (200 nM) for 4 h. 53BP1 foci were analyzed in Cyclin A-negative cells after the removal of 4-OHT (time 0 h).

After immunostaining of cells, foci were visualized by confocal microscopy (SP8) and immunofluorescence microscopy (BZ-9000, KEYENCE). We counted the number of subnuclear foci in at least 50 G_1_-phase (Cyclin A-negative) cells per experiment.

#### Detection of TOP2ccs in genomic DNA

To measure the covalently associated TOP2 with genomic DNA, chromatin were extracted from 4 million cells (TK6 and MCF-7) After genomic fragmentation by sonication (UR-21P) (6× (30 s) at power 8), the chromatin extract (2 mL) was then subjected to ultracentrifugation at 100,000 ×*g* for 16 h at 25°C in a cesium chloride gradient (1.45, 1.5, 1.7 and 1.86 g/mL; 2 mL each).

A total of 1 mL was collected for analysis from the top to bottom of the cesium chloride gradient. For the slot blot analysis, 100 μL of each of the collected fractions were spotted onto methanol-pretreated PVDF membrane through the slot of Bio-Dot apparatus. After brief washing with 0.2 M phosphate buffer (pH 6.8), the membrane was incubated overnight at 4°C with anti-TOP2β (1:2,000) diluted in 5% skimmed milk in TBST (0.01 M Tris-HCl, 150 mM NaCl, 0.005% Tween 20, pH 8) followed by incubation with horseradish peroxidase (HRP)-conjugated anti-mouse secondary antibody (1/5000) for 1 h at room temperature. Immunoreactive spots were developed by chemiluminescence using the ECL reagent (ECL^™^ Prime western blotting detection reagent). The signal was detected by exposure to X-ray film (Amersham Hyperfilm^™^ MP) and scanned (EPSON) for quantification by ImageJ software. The protocol is adapted from ref.^49^

#### Western blot analysis

MCF-7 and TK6 cells (5×10^5^) in 50 μL of PBS were lysed by the addition of 50 μL 2× lysis buffer (120 mM Tris-HCl, 4% SDS, 0.04% bromophenol blue, 10% glycerol, and 10% 2-mercaptoethanol). After boiling the samples for 10 min, lysates were briefly centrifuged and a sample (10 μL) from 100 μL of the supernatant was separated by polyacrylamide gel electrophoresis (5%–10% gel for ATM or 5%–20% for TDP2) for several hours at 200 mV. These separated proteins were then transferred into a nitrocellulose membrane (for ATM) and PVDF membrane (for TDP2) using the semidry method. Membranes were blocked with 5% BSA (for ATM) or 5% skimmed milk (for TDP2) in TBST at room temperature for >1 h prior to incubation with primary antibody overnight at 4°C. The membrane was washed several times with TBST solution and then incubated with an appropriate HRP-linked secondary antibody (1:5,000, in blocking buffer) for 1 h at room temperature. After washing (×3), immunoreactive bands were developed by chemiluminescence using ECL reagent (ECL^™^ Prime western blotting detection reagent). The signal was detected by exposure to X-ray film (Amersham Hyperfilm^™^ MP) and scanned by a scanner (EPSON). Details of the antibodies used are listed in Key resources table.

#### Cell survival assay

For TK6 cells, the cell survival assay was performed according to the method described by ref.^49^ In brief, various amounts of etoposide were mixed with 1.5% (w/v) methylcellulose in medium (RPMI) containing 10% horse serum, by slowly rotating tubes overnight at 4°C. Fixed numbers of cells were seeded into 6-well plates containing 5 mL methylcellulose medium per well, and incubated for 2 weeks at 37°C before counting visible colonies.

For MCF-7 cell, fixed numbers of cells (500–2,000) were cultured in 10% FBS containing DMEM and then incubated at 37°C with various amount of etoposide for 2–3 weeks. After washing with dH_2_O, plates were stained with 5% Giemsa solution to visualize and count the colonies.

#### Transcriptome analysis by NET-CAGE

MCF-7 cells were cultured in a serum-free medium for 24–26 h before treatment with 10 nM E2. To assess the effect of ATM loss on the E2 response, 10 μM of ATMi was added to the medium together with E2 or ethanol; DMSO was added 30 min prior to the E2 treatment as a control. Three replicates of cells cultured in three 15-cm dishes (approximately 10^6^ cells per dish) were treated simultaneously and pooled for analysis. Replicates were processed simultaneously throughout to avoid potential batch effects. RNA was extracted as described previously.^76^ Briefly, fresh cells were lysed in the presence of α-amanitin, and the nuclear insoluble fraction was isolated. Following DNase treatment, RNA was purified using the miRNeasy Mini kit (Qiagen) according to the manufacturer’s instructions. NET-CAGE library preparation, sequencing, mapping, and gene expression analysis were performed by DNAFORM (Kanagawa, Japan). In brief, the cDNAs were synthesized from total RNA using random primers. The ribose diols in the 5′ cap structures of RNAs were oxidized and then biotinylated. The biotinylated RNA/cDNAs were selected by streptavidin beads (cap-trapping). After RNA digestion by RNaseONE/H and adaptor ligation to both cDNA ends, double-stranded cDNA libraries (CAGE libraries) were constructed. CAGE libraries were sequenced using single-end reads of 75 nt on a NextSeq 500 instrument (Illumina). Obtained reads (CAGE tags) were mapped to the human hg38 genome using BWA (version 0.5.9) (https://arxiv.org/abs/1303.3997). Unmapped reads were then mapped by HISAT2 (version 2.0.5).^138^ CAGE tag count data were clustered through the CAGEr toolbox^139^ using Paraclu algorithm^140^ with default parameters. Clusters with count per million (CPM) < 0.2 were discarded. Differentially expressed genes at each time point were detected using DESeq2 (version 1.20.0).^141^ Differential temporal expression patterns in time course data were detected and clustered using TCseq (https://www.bioconductor.org/packages/release/bioc/html/TCseq.html) with default parameters. The mapping statistics are listed in Table S2. DHS data^142^ and H3K27ac and H3K4Me1 data^109^ were used for heatmap generation.

#### Other datasets

We used ER and FOXA1 binding site data obtained from Cistrome^143^ based on ChIP-seq in MCF-7 (GEO accession numbers GSE68359 and GSE80808, respectively).^144,145^

#### mRNA quantification

MCF-7 and T47D cells were cultured in DMEM containing 10% FBS and then cultured in a serum-free media for 24 h. After washing with cold PBS cells were collected for RNA isolation. RNA was isolated by the Sepasol, chloroform, and 2-propanol method. Total RNA (500 ng) was used for cDNA synthesis with the PrimeScript^™^ first strand cDNA synthesis kit. Synthesized cDNA was diluted with sterilized MilliQ water (1/50 dilution) and then analyzed by digital PCR.

#### Chromatin immunoprecipitation (ChiP)

The G1 phase (24-h serum starved) MCF-7 cells were first fixed with 1% formaldehyde for 10 min and then quenched with 2.5M glycine solution for 5 min at room temperature. Chromatin extracts were sonicated (UR-21P) (6× (30 s) at power 8) to generate DNA fragments (<500 bp). Sheared chromatin was centrifuged at 15,000 rpm for 15 min at 4°C and after centrifugation, super-natants were incubated with α-γH2AX antibody (Key resources table) and Dynabeads Protein A at 4°C for overnight. The conjugated beads were washed thoroughly with IP buffer-140, IP buffer-500, LiCl/detergent, and TE. Real-time PCR was carried out using THUNDERBIRD SYBR qPCR Mix. Primer sequences were listed in Table S1. The protocol is adapted from a previous study.^60^

#### Chromosome conformation capture (3C)

3C analysis was conducted by following (epigenome-noe.net/researchtools/protocol.php_protid = 6.html#reagentss) with slight modifications. In brief, serum-starved MCF-7 cells treated with 100 nM E2 and 10 μM ATMi or vehicle control (DMSO for ATMi and EtOH for E2) were fixed with 0.5% formalin for 10 min and then quenched with 0.125 M glycine for 5 min at room temperature. Approximately 10^6^ cells were centrifuged (800 ×g for 10 min), dissolved with 0.9% SDS containing 1× NEBuffer^™^ r3.1 buffer and then incubated with SDS (0.3% final) at 37°C for 1 h. SDS was then quenched by incubation in 1.8% Triton X-100 at 37°C for 1 h. The chromatin was then digested with *Bgl*II (600 u) for 20 h. After inactivating the restriction enzyme at 65°C, digested chromatin was then ligated for 4 h in a total reaction volume of 800 μL consisting of 200 μL digested chromatin (from 800 μL) and using 0.0375 u/μL T4 DNA ligase (NEB). Chromatins were then de-crosslinked by incubation with proteinase K (100 μg/mL final) at 65°C overnight and genomic DNA was purified by the phenol-chloroform extraction method.

The restriction enzyme digestion and ligation were then assessed by agarose gel (0.8%) electrophoresis. Ligated genomic DNA (300 ng) was analyzed by Touched Down PCR under the following conditions: 95°C for 2 min followed by (5 cycles of 95°C for 10 s, 68°C for 30 s, and 72°C for 30 s), (5 cycles of 95°C for 10 s, 66°C for 30 s, and 72°C for 30 s), (5 cycles of 95°C for 10 s, 64°C for 30 s, and 72°C for 30 s), (5 cycles of 95°C for 10 s, 62°C for 30 s, and 72°C for 30 s), (15 cycles of 95°C for 10 s, 58°C for 30 s, and 72°C for 30 s), with a final incubation at 72°C for 4 min. To quantify The interaction between promoter and enhancer a nested qPCR was performed under the following conditions: 95°C for 5 min followed by 40 cycles of 95°C for 15 s, 60°C for 30 s, and 72°C for 45 s. A sample of the first PCR product (1:100) was used as the template. Primer sequences are given in Table S1.

#### CRISPR-based enhancer cleavage

To analyze the effect of CRISPR-induced breaks in the *c-MYC* enhancer, the CRISPR-cas9 and gRNA vector was introduced into cells by lentiviral infection. Cells were then serum-starved for 24 h (along with the virus infection) and treated with 10 nM E2 for 30 min. After RNA purification (by the same method described for mRNA quantification), cDNA was synthesized by ReverTra Ace ^®^ qPCR RT Master Mix (Toyobo). The cDNA was then diluted with sterilized MilliQ water (1:20) for quantitative PCR (qPCR) analysis using the THUDERBIRD^™^ SYBR^®^ qPCR Mix. Signals were detected by StepOnePlus real-time PCR system with StepOne software ver2.2.2.

### QUANTIFICATION AND STATISTICAL ANALYSIS

#### Statistical analysis

Data are presented as mean ± SD (standard deviation) or median + inter-quartile ranges as indicated in the figure legends. Unpaired Student’s t-tests from at least 3 biological replicates were performed using Microsoft Excel (Microsoft Corporation).

## Supplementary Material

1

## Figures and Tables

**Table:** KEY RESOURCES TABLE

REAGENT or RESOURCE	SOURCE	IDENTIFIER
Antibodies		
Rat monoclonal α-Cytokeraitn-8	Developmental Studies Hybridoma Bank	Cat# TROMA-I;RRID: AB_531826
Rabbit polyclonal α−53BP1	EMD millipore corp.	Cat# Pc712; RRID: AB_564982
Rabbit polyclonal α-cMYC	Abcam	Cat # ab32072; RRID: AB_731658
α-PCNA, clone PC10 conjugated to Alexa Fluor 647	BioLegend	Cat# 307912; RRID: AB_2267947
Mouse monoclonal α-γH2Ax	EMD millipore	Cat# 05–636;RRID: AB_309864
Rabbit polyclonal α-CyclinA (clone C19)	Santa Cruz	Cat# sc-596; RRID: AB_631330
Mouse monoclonal α-β-actin	Sigma	Cat# A5411; RRID: AB_2766219
Mouse monoclonal α-ATM (5C2)	Novus Biologicals	Cat# NB100–220; RRID: AB_2274595
Rabbit polyclonal α-TOP2B	Novus Biologicals	Cat# NB100–40842; RRID: AB_792364
Rabbit polyclonal α-TDP2	Bethyl	Cat# A302–737A; RRID: AB_10631698
Alexa Fluor^™^ 488 goat α-rat	Invitrogen	Cat# A11006; RRID: AB_141373
Alexa Fluor^™^ 594 goat a-rabbit	Invitrogen	Cat# A11036; RRID: AB_10563566
Goat polyclonal α-mouse HRP	Thermo Fisher	Cat# 32430; RRID: AB_1185566
Donkey polyclonal α-rabbit HRP	Santa Cruz	Cat# sc-2313, RRID: AB_641181
Chemicals, peptides, and recombinant proteins		
Charcoal/Dextran treated FBS	Hyclone Laboratories	Cat#SH30068.03
Giemsa’s Stain Solution	Nacalai Tesque	Cat# 377114–35
Albumin, Bovine, F-V, pH5.2	Nacalai Tesque	Cat# 01863–48
Skim Milk for immunoassay	Nacalai Tesque	Cat# 31149–75
MG132	Wako	Cat# 135–18453
17β-Estradiol solution	Sigma	Cat# E–060
KU-55933 (ATMi) (ATM Kinase Inhibitor)	Selleckchem.com	Cat #S1092
NU7441(DNA-PKi) (DNA-PK inhibitor)	Selleckchem.com	Cat #S2638
cMYCi (KJ-Pyr-9)	Namiki Shoji	# HY-19735
Polyethylene Glycol (PEG) # 300	Nacalai Tesque	#28214–05
EdU	Invitrogen	# A10044
Alexa Fluor^®^ 647 Azide	Invitrogen	#A10277
Optical cutting temperature (OCT) compound	Sakura Finetek Japan	Cat# 4583
Cryomold	Sakura Finetek Japan	Cat# 4665
Paraformaldehyde	Nacalai Tesque	#09154–14
Formaldehyde solution (Formalin)	Sigma-Aldrich	#F1635
DAPI	Nacalai Tesque	#12745–74
Hoechst 33,342,346–07951	Dojindo	# 346–07951
RPMI 1640	Nacalai Tesque	# 3026456
Horse serum	Gibco	# 16050–122
Penicillin/Streptomycin	Nacalai Tesque	# 09367–34
Sodium pyruvate	Sigma	S8636
L-glutamate	Nacalai Tesque	#16948–04
Fetal Bovine Serum (FBS)	Gibco	10,270–106
DMEM	Nacalai Tesque	#08459–64
Bio-Dot apparatus		#84BR23017
ECL reagent (ECL^™^ Prime)	Cytiva	# RPN2232
X-ray film (Amersham Hyperfilm^™^ MP)	Cytiva	# 28906850
Dynabeads Protein G Immunoprecipitation	Thermo Fischer	Cat# 10003D
Protease inhibitor cocktail, Complete	Sigma	Cat#11697498001
Lipofectamine 3000 Transfection Kit	Thermo Fischer	Cat# L3000008
FuGENE HD Transfection Reagent	Promega	Cat# E2312
Doxycycline Hydrochloride	MP Biomedicals, Inc.	Cat# 195044
(Z)-4-Hydroxytamoxifen	Sigma	Cat# H7904
Fulvestrant	Sigma	Cat# I4409
3′-Indoleacetic Acid	Nacalai Tesque	Cat# 19119–61
Etoposide	Trevigen	Cat# 4886-400-01
α-Amanitin	Sigma	Cat# A2263
2.5 g/L-Tripsin/1 mmol/L-EDTA Solution	Nacalai Tesque	Cat# 35554–64
Sepasol-RNA I Super G	Nacalai Tesque	# 09379–97
T4 DNA ligase	NEB	#M0202S
Critical commercial assays		
GeneArt Seamless Cloning Enzyme Mix ThermoFischer Cat# A14606	Thermo Fisher Scientific	Cat# A14606
PrimeScript^™^ 1^st^ strand cDNA synthesis kit	Takara	# 6210A
THUDERBIRD^™^ SYBR^®^ qPCR Mix	Toyobo	# QPS-201
ReverTra Ace^®^ qPCR RT Master Mix	Toyobo	FSQ-301S
cMYC Digital PCR mix	Thermo Fisher Scientific	#Hs00153408_m1
TFRC Digital PCR mix	Thermo Fisher Scientific	#Hs00951083_m1
miRNeasy Mini Kit	QIAZEN	#217004
Experimental models: Cell lines		
Human: MCF-7 WT	ATCC	Cat# HTB-22
Human: TK6 (TSCER2) WT	A gift from Dr. Masamitsu Honma	N/A
Human: Lenti-X^™^ 293T	TAKARA	Cat# 632180
Human: T47D WT	ATCC	HTB-133
Human, MCF-7, *ATM*^−/−^	This study	N/A
Human, MCF-7, *TOP2B* ^−/−^	Sasanuma et al., 2018^60^	N/A
Human, MCF-7, *TDP2* ^−/−^	This study	N/A
Human, MCF-7, *TDP2*^−/−^ TDP2	This study	N/A
Human, MCF-7, *TDP2*^−/−^/dTDP2	This study	N/A
Human, MCF-7, *TDP2*^−/−^/+Mock	This study	N/A
Human, TK6, *LIG4*^−/−^	Akagawa et al., 2020^53^	N/A
Human, TK6, *ATM*^−/−^	This study	N/A
Human, TK6, *DNA-PKcs*^−/−^	Akagawa et al., 2020^53^	N/A
Human, TK6, *CtIP*^*T847/859A*^	This study	N/A
Human, TK6, *ATM*^−/−^*CtIP*^*T847/859E*^	This study	N/A
Human, TK6, *CtIP*^*T847/859A*^*/CtIP*^*T847/859E*^	This study	N/A
Human, TK6, *CtIP*^*AID/AID*^	Hoa et al., 2015^135^	N/A
Oligonucleotides		
MCF-7, *ATM* (for gene disruption) gRNA #1: 5’-ATATGAACACGAAGCAATGT-3’	This study	N/A
MCF-7, *ATM* (for gene disruption) gRNA#2: 5’-AATCCCCTCATCAACACGCC −3’	This study	N/A
MCF-7, *ATM* (for gene disruption) gRNA #3: 5’-GAAAAAAGTAAAGAAGAAAC-3’	This study	N/A
MCF-7, *TDP2* (for gene disruption), 5’-GGCTCAGAGATGGTTTCAGGT-3’	This study	N/A
MCF-7, *TOP2B* (for gene disruption), 5’-CGGCGTGGGCGGCGGCAACG −3’	This study	N/A
TK6, *ATM* (for gene disruption) 5’-AATCCCCTCATCAACACGCC-3’	This study	N/A
TK6, *CtIP* ^*T847/859A*^, (Knock-In) gRNA #1: 5’-CTAAGATATTCAGCAGTCTA-3’	This study	N/A
TK6, *CtIP*^*T847/859A*^, (Knock-In) gRNA#2: 5’-CAAATATCGACIIIIIIICC-3’	This study	N/A
Oligonucleotide continued in Table S1	N/A	N/A
Recombinant DNA		
Plasmid: lentiCRISPRv2-puro	Addgene	#98290
Plasmid: pX330	Addgene	#42230
Plasmid: pX459	Addgene	#48139
Plasmid: pSpCas9(BB)-2A-GFP (PX458)	Addgene	#48138
Plasmid: lentiCRISPRv2-AsiSI	A gift from Tanya Paull	N/A
Plasmid: pTP2630, CtIP^T847/859A^ cDNA	A gift from Tanya Paull	N/A
Plasmid: pTP3890, CtIP^T847/859E^ cDNA	A gift from Tanya Paull	N/A
Plasmid: pSINDUAL TDP2	Cortes Ledesma et al., 2009^55^	N/A
Plasmid: pSINDUAL TDP2-catalytic dead	Cortes Ledesma et al., 2009^55^	N/A
Plasmid: lentiCRISPRv2-empty	This study	N/A
Plasmid: lentiCRISPRv2-TDP2	This study	N/A
Plasmid: lentiCRISPRv2-dTDP2	This study	N/A
Software and algorithms		
BWA (version 0.5.9)	https://arxiv.org/abs/1303.3997	N/A
HISAT2 (version 2.0.5)	Kimetal., 2015^138^	N/A
CAGEr toolbox	Haberle et al., 2015^139^	N/A
Paraclu algorithm	Frith etal.,2008^140^	N/A
DESeq2 (version 1.20.0)	Loveetal., 2014^141^	N/A
TCseq	https://www.bioconductor.org/packages/release/bioc/html/TCseq.html	N/A
Deposited data		
Raw and processed NET-CAGE data	This study; GEO	GEO: GSE218320
Raw data except for the NET-CAGE data	This study; Mendeley Data	https://doi.org/10.17632/v74k5sntdk.1a

## Highlights

- ATM promotes the repair of DNA TOP2-dependent DNA breaks
- Unrepaired TOP2-dependent DSBs impair early transcriptional responses to estrogens
- TOP2 generates a DNA break at the *c-MYC* enhancer after estrogen exposure
- Loss of ATM results in *c-MYC* gene overexpression after estrogen exposure

## INCLUSION AND DIVERSITY

We support inclusive, diverse, and equitable conduct of research.
