# Supplementary material for: ATM suppresses c-Myc overexpression in the mammary epithelium in response to estrogen
Source: Cell Rep. Author manuscript; Available in PMC 2023 Mar 17. (PMC10023214; doi:10.1016/j.celrep.2022.111909)
Supplement: 1 [file NIHMS1870498-supplement-1.pdf]

Cell Reports, Volume *42*

Supplemental information

ATM suppresses c-Myc overexpression in the mammary epithelium in response to estrogen

Rifat Ara Najnin, Md Rasel Al Mahmud, Md Maminur Rahman, Shunichi Takeda, Hiroyuki Sasanuma, Hisashi Tanaka, Yasuhiro Murakawa, Naoto Shimizu, Salma Akter, Masatoshi Takagi, Takuro Sunada, Shusuke Akamatsu, Gang He, Junji Itou, Masakazu Toi, Mary Miyaji, Kimiko M. Tsutsui, Scott Keeney, and Shintaro Yamada

Figure S1

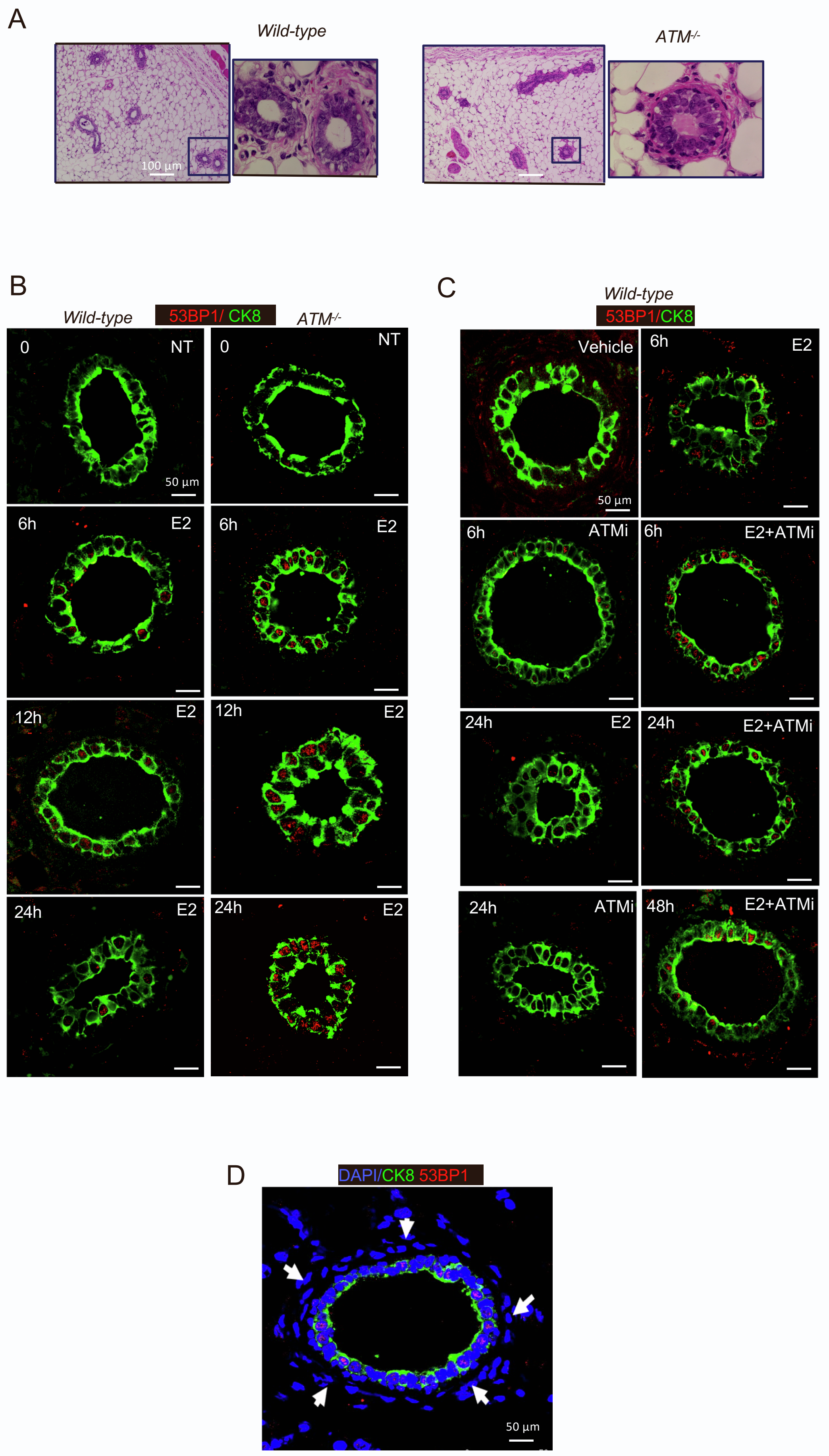
A
Wild-type
ATM-/-
100 µm
B
Wild-type
53BP1/ CK8
ATM-/-
0
NT
50 µm
6h
E2
12h
24h
C
Wild-type
53BP1/CK8
Vehicle
ATMi
E2+ATMi
48h
D
DAPI/CK8 53BP1
50 µm

Figure S1. Estrogen (E2) is genotoxic to mammary epithelial cells in Atm-deficient mice, related to Figure 1

(A) H&E staining of *wild-type* and *ATM*$^{-/-}$ B6;129 mouse mammary gland analyzed in Figure 1C. Scale bar represents 100 μm.

(B) Representative image of 53BP1^{+} epithelial cells at 6 h after i.p. injection of E2 into B6 mice carrying the indicated genotype. Mammary glands were isolated at 0, 6, 12, and 24 h after i.p. injection and immunostained with α-53BP1and α-CK8-specific antibodies. Quantification is shown in Figure 1C.

(C) Representative image of 53BP1^{+} epithelial cells in *wild-type* B6 mice after i.p. injection of E2 and ATMi. Mammary glands were isolated at 6, 24, and 48 h after i.p. injection and immunostained with α-53BP1- and α-CK8-specific antibodies. Quantification is shown in Figure 1D.

(D) CK8 and 53BP1 negative non-epithelial stromal cells are indicated by white arrows.

(B-D) Scale bar represents 50 μm.

Figure S2

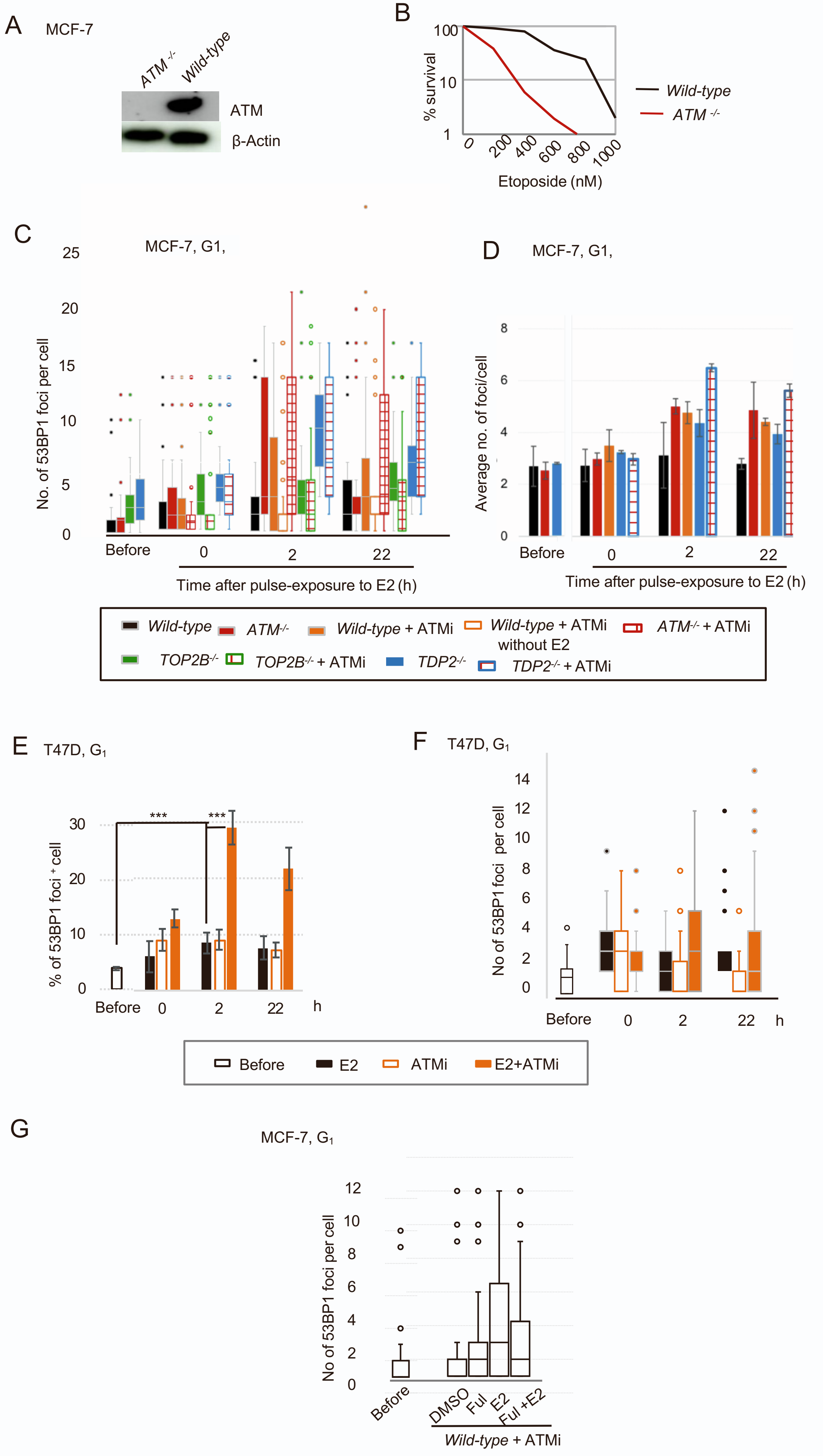
A
MCF-7
ATM -/-
Wild-type
ATM
β-Actin
B
100
10
1
% survival
0
200
400
600
800
1000
Etoposide (nM)
Wild-type
ATM -/-
C
MCF-7, G1,
No. of 53BP1 foci per cell
25
20
15
10
5
0
Before
0
2
22
Time after pulse-exposure to E2 (h)
D
MCF-7, G1,
Average no. of foci/cell
8
6
4
2
0
Before
0
2
22
Time after pulse-exposure to E2 (h)
Wild-type
ATM-/-
Wild-type + ATMi
Wild-type + ATMi without E2
ATM-/- + ATMi
TOP2B-/-
TOP2B-/- + ATMi
TDP2-/-
TDP2-/- + ATMi
E
T47D, G1
% of 53BP1 foci + cell
30
20
10
0
***
***
Before
0
2
22
h
F
T47D, G1
No of 53BP1 foci per cell
14
12
10
8
6
4
2
0
Before
0
2
22
h
Before
E2
ATMi
E2+ATMi
G
MCF-7, G1
No of 53BP1 foci per cell
12
10
8
6
4
2
0
Before
DMSO
Ful
E2
Ful +E2
Wild-type + ATMi

Figure S2. ATM promotes the repair of E2-induced TOP2-dependent DSBs in human breast cancer cells, related to Figure 2

(A) Western blot analysis to detect ATM and β-Actin (loading control) in MCF-7 cells.

(B) Colony survival of the indicated MCF-7 cells. The x- and y-axes show the concentration of etoposide in a linear scale and % survival in a log scale, respectively.

(C) Box plot showing the number of 53BP1 foci per cell (MCF-7). The data from one individual experiment of Figure 2C are shown here.

(D) Average number of 53BP1 foci per cell in the indicated genotypes. Data are replotted from the same experiments of Figure 2C and represent the mean ± SD, from three independent experiments.

(E) Percentage of G_1-phase $53BP1^+$ T47D cells (≥5 foci per cell) carrying the indicated genotypes. Data represent the mean ± SD, calculated from three independent experiments. ***$P < 0.005$, unpaired two-tailed Student's *t*-test.

(F) The data (T47D) is shown as in (C). The data from one individual experiment of Figure S2E are shown here.

(G) The data (MCF-7) is shown as in (C). The data from one individual experiment of Figure 2D are shown here.

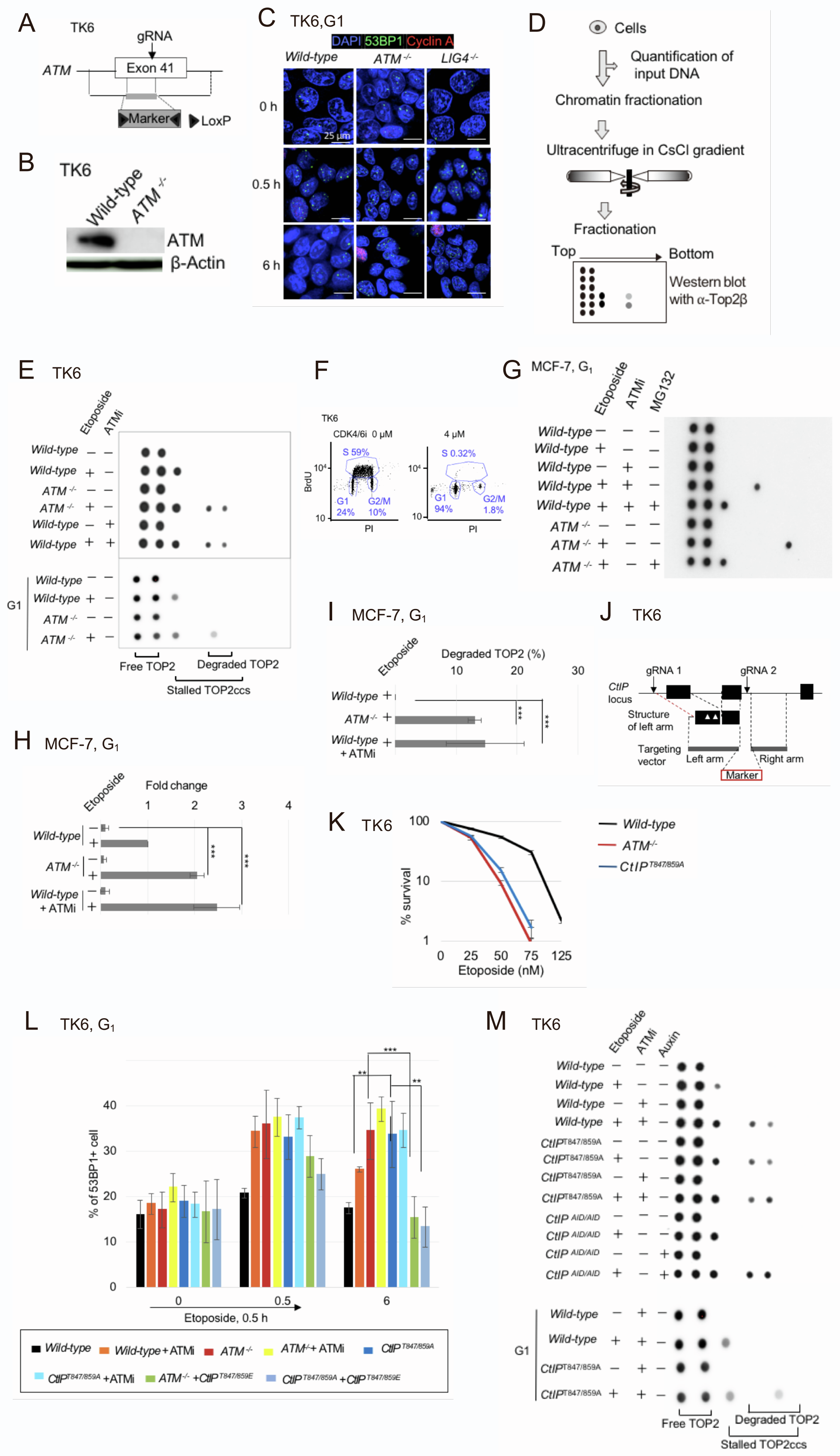
A
TK6
gRNA
ATM
Exon 41
Marker
LoxP
B
TK6
Wild-type
ATM -/-
ATM
β-Actin
C
TK6,G1
DAPI 53BP1 Cyclin A
Wild-type
ATM -/-
LIG4 -/-
0 h
0.5 h
6 h
25 µm
D
Cells
Quantification of input DNA
Chromatin fractionation
Ultracentrifuge in CsCl gradient
Fractionation
Top
Bottom
Western blot with α-Top2β
E
TK6
Etoposide
ATMi
Wild-type
ATM -/-
G1
Free TOP2
Degraded TOP2
Stalled TOP2ccs
F
TK6
CDK4/6i 0 µM
4 µM
S 59%
S 0.32%
G1 24%
G2/M 10%
G1 94%
G2/M 1.8%
BrdU
PI
G
MCF-7, G1
Etoposide
ATMi
MG132
Wild-type
ATM -/-
H
MCF-7, G1
Etoposide
Fold change
Wild-type
ATM -/-
Wild-type + ATMi
I
MCF-7, G1
Etoposide
Degraded TOP2 (%)
Wild-type
ATM -/-
Wild-type + ATMi
J
TK6
gRNA 1
gRNA 2
CtIP locus
Structure of left arm
Targeting vector
Left arm
Right arm
Marker
K
TK6
% survival
Etoposide (nM)
Wild-type
ATM -/-
CtIP T847/859A
L
TK6, G1
% of 53BP1+ cell
Etoposide, 0.5 h
Wild-type
Wild-type+ATMi
ATM -/-
ATM -/- + ATMi
CtIP T847/859A
CtIP T847/859A +ATMi
ATM -/- +CtIP T847/859E
CtIP T847/859A +CtIP T847/859E
M
TK6
Etoposide
ATMi
Auxin
Wild-type
CtIP T847/859A
CtIP AID/AID
G1
Free TOP2
Degraded TOP2
Stalled TOP2ccs

Figure S3. ATM promotes the removal of 5' TOP2 adducts from DSB ends in the G_1 phase by phosphorylating CtIP at T847/T859, Related to Figure 3

(A) The *ATM*-gene disruption construct and the site recognized by guide RNA (gRNA) in TK6 cells. Exon 41 contains the catalytic site.

(B) Western blot analysis of ATM expression using β-Actin as a reference.

(C) Representative 53BP1 foci in the indicated cells before and after pulse exposure (0.5 h) to etoposide and after 5.5 h repair time. Blue, green, and red indicate DAPI, 53BP1, and cyclin A signals, respectively. Figure 3A shows the quantification of $53BP1^+$ cells. Scale bar represents 25 μm.

(D) The measurement of stalled TOP2ccs by immunodetection with an α-TOP2 antibody.

(E) Representative dot blot of TOP2ccs for Figure 3F and 3G. Genomic DNA from *wild-type* cells treated with etoposide for 2 h was included as a control for every dot blot. The first and second fractions represent free TOP2. The third fraction contains stalled TOP2ccs having intact TOP2. The fifth and sixth fractions contain stalled TOP2ccs with degraded TOP2. 'G_1' indicates TK6 cells arrested in the G_1 phase (Figure S3F). Quantification is shown in Figure 3F for the whole stalled TOP2ccs and in Figure 3G for stalled TOP2ccs with degraded TOP2. Cesium chloride gradient ultracentrifugation fractionates proteins, protein-DNA complexes, and DNA by their differential specific gravities, resulting in the enrichment of free TOP2 (light), TOP2-DNA complexes (intermediate), and DNA (heavy) in the top, middle, and bottom fractions, respectively. The specific gravity of DNA covalently associated with partially proteolyzed TOP2 adducts is higher (heavier) than that with intact TOP2 adducts; therefore, the former is more enriched in the lower fractions than the latter. This is further supported by our data (Figure S3G) showing that the proteasome inhibitor MG132 shifts the TOP2-DNA complex signal from the lower fifth fraction to the upper (lighter) third fraction as we reported previously.[S1]

(F) The frequency of TK6 cells treated with CDK4/6i (Palbociclib) for 48 h indicating the enrichment of G_1 phase cells.

(G) Representative dot blot of TOP2ccs in G_1 phase MCF-7 cells for Figure S3H and S3I. The addition of a proteasome inhibitor (MG132) caused the shift of signals from the middle fractions (from the top, lanes 4, 7) to the upper-third fraction (lanes 5, 8).

(H) Quantification of the whole stalled TOP2ccs shown in the western blot shown in Figure S3G. Data are presented as in (F).

(I) Quantification of stalled TOP2 having degraded TOP2 shown in the western blot shown in Figure S3G. The x-axis shows the percentage of amounts of degraded TOP2ccs relative to amounts of whole stalled TOP2ccs in (H).

(J) Schematic diagram of *CtIP* $^{T847A/T859A}$ mutation knockin into exon 18, using the indicated targeting vector. The white triangles show the T847A and T859A mutations. The left arm of the knockin construct consists of exons 18 and 19. Cells were transiently co-transfected the indicated gRNA expression vectors with the knockin construct.

(K) Colony survival of the indicated genotypes of TK6 cells. The data are presented as in Figure S2B.

(L) The repair kinetics of etoposide-induced breakage expressed as the percentage of 53BP1 foci^{+} cells (≥ 5 foci). Cells were treated with etoposide for 30 min. ATMi was added 30 min prior to the etoposide treatment until the 53BP1 foci were counted.

(M) Representative dot blots of TOP2ccs for the indicated TK6 cells. Experiments were performed as in Figure 3D. Quantification is shown in Figure 3H and 3I.

(H, I, K, and L) Data represent the mean ± SD, from three independent experiments. $^{**}P < 0.05$, $^{***}P < 0.005$, Student's *t*-test.

Figure S4

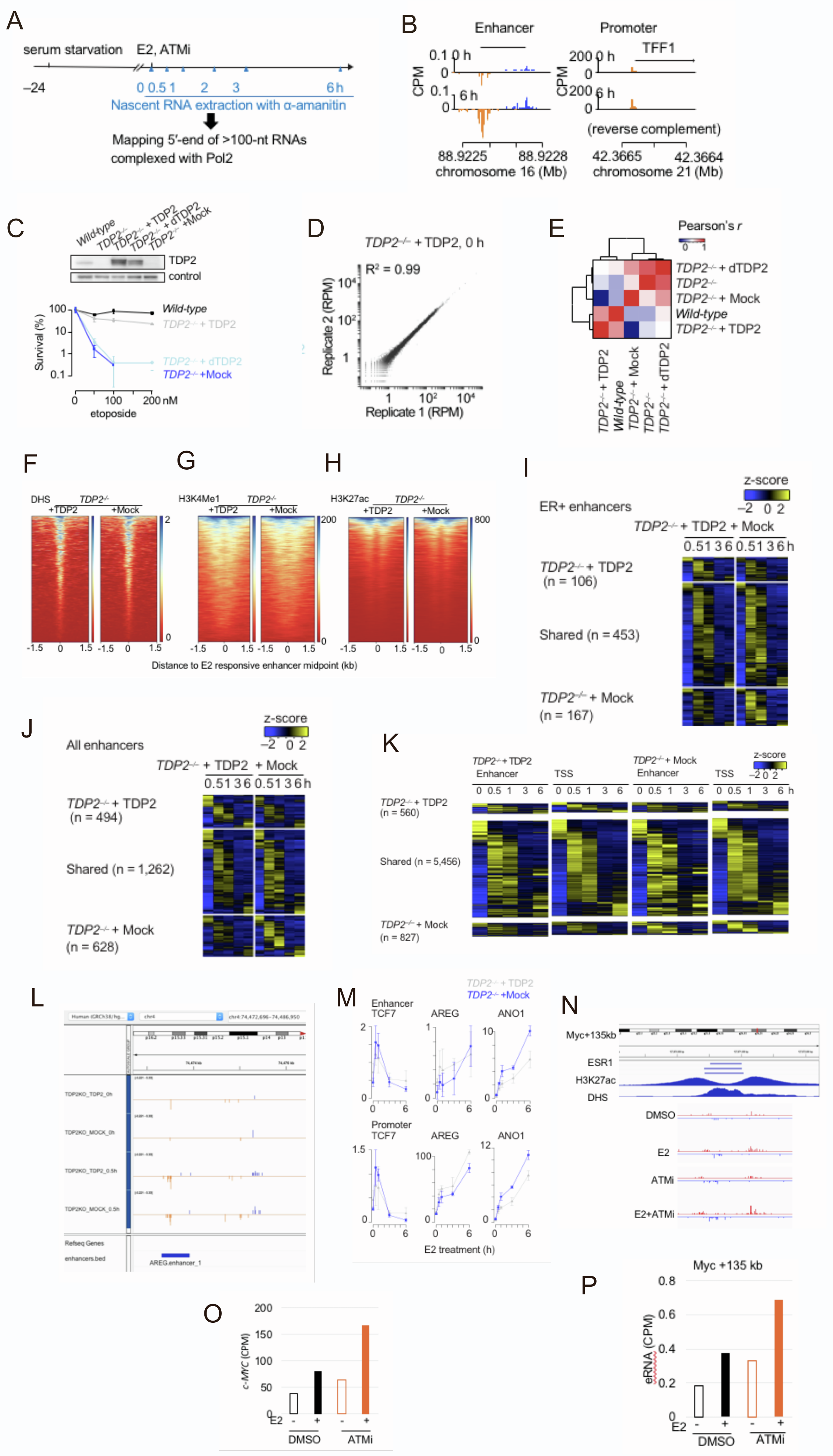

A
serum starvation
E2, ATMi
–24
0 0.5 1 2 3 6 h
Nascent RNA extraction with α-amanitin
Mapping 5′-end of >100-nt RNAs complexed with Pol2
B
Enhancer
Promoter
TFF1
CPM
0 h
6 h
(reverse complement)
88.9225 88.9228
chromosome 16 (Mb)
42.3665 42.3664
chromosome 21 (Mb)
C
Wild-type
TDP2-/-
TDP2-/- + TDP2
TDP2-/- + dTDP2
TDP2-/- +Mock
TDP2
control
Survival (%)
etoposide
D
TDP2-/- + TDP2, 0 h
R² = 0.99
Replicate 2 (RPM)
Replicate 1 (RPM)
E
Pearson's r
F
DHS
G
H3K4Me1
H
H3K27ac
Distance to E2 responsive enhancer midpoint (kb)
I
ER+ enhancers
z-score
TDP2-/- + TDP2 (n = 106)
Shared (n = 453)
TDP2-/- + Mock (n = 167)
J
All enhancers
TDP2-/- + TDP2 (n = 494)
Shared (n = 1,262)
TDP2-/- + Mock (n = 628)
K
Enhancer
TSS
TDP2-/- + TDP2 (n = 560)
Shared (n = 5,456)
TDP2-/- + Mock (n = 827)
L
TDP2KO_TDP2_0h
TDP2KO_MOCK_0h
TDP2KO_TDP2_0.5h
TDP2KO_MOCK_0.5h
Refseq Genes
enhancers.bed
AREG.enhancer_1
M
Enhancer
Promoter
TCF7
AREG
ANO1
E2 treatment (h)
N
Myc+135kb
ESR1
H3K27ac
DHS
DMSO
E2
ATMi
E2+ATMi
O
c-MYC (CPM)
E2
DMSO
ATMi
P
Myc +135 kb
eRNA (CPM)
E2
DMSO
ATMi

Figure S4. Defective repair of stalled TOP2ccs dysregulates estrogen-dependent activation of potential enhancers, Related to Figure 4

(A) Schematic diagram of NET-CAGE time-course experiments shown in Figure 4. MCF-7 cells were cultured in serum-free medium for 24 h and stimulated with E2. We purified RNAs complexed with Pol2 and >100 nt in length in the presence of the Pol2 inhibitor α-amanitin, excluding short RNAs derived from paused Pol2. The 5′ end of nascent RNAs was analyzed by CAGE RNA sequencing.

(B) A genome browser view of NET-CAGE data for an enhancer and a promoter. The y axes represent counts per million (CPM). Reads mapped on the top and bottom strands were counted at the 5′ end of nascent RNA and shown in orange and blue, respectively. Note that transcripts were produced bi-directionally from the enhancer, unlike those from the promoter.

(C) Western blot analysis of TDP2 expression (top) and etoposide sensitivities (bottom) of the indicated MCF-7 cells. The sensitivities are presented as in Figure S2B.

(D) Reproducibility (R^2, coefficient of determination) of NET-CAGE maps. NET-CAGE transcript tag counts from biological replicates from *TDP2*$^{-/-}$ + TDP2 cells harvested at 0 h were summed per gene. Each point represents one gene.

(E) A heatmap showing Pearson correlation coefficient (Pearson's *r*) based on fold changes (1 h/0 h) in expression of E2 responsive genes.

(F, G, H) Heat maps of DHS, histone 3 lysine 4 mono-methylation (H3K4me1) and H3K27ac signals ± 2 kb around estrogen-responsive enhancers identified by NET-CAGE.

(I-J) Heat maps show eRNA profiles of E2-responsive enhancers located within 1 kb of known ERα binding sites (ER+ enhancers, F) or all E2-responsive enhancers (all enhancers, G). Heat maps showing log fold change ($\log_2$) of eRNAs at the indicated time relative to those at 0 h after exposure of MCF-7 cells to E2. Enhancers were classified as: enhancers that significantly expressed eRNAs either only in *TDP2*$^{-/-}$ + TDP2 cells (top), only in *TDP2*$^{-/-}$ + Mock (bottom), or in both (middle) in response to E2 exposure.

(K) Heat maps showing the simultaneous response to E2 exposure between all the possible pairs of TSSs and neighboring (<400 kb) enhancers. Heat maps showing log fold change ($\log_2$) of all enhancer and TSS expression levels at the indicated time relative to those at 0 h after exposure of MCF-7 cells to E2. Enhancers were classified as in (I).

(L) A genome browser view of NET-CAGE data for an E2 responsive neighboring gene, AREG enhancer in *TDP2*$^{-/-}$ + TDP2 and *TDP2*$^{-/-}$ + Mock cells comparing without (0h) and with (0.5h) E2 treatment.

(M) Correlation of E2 response between a neighboring enhancer and a TSS at example genes.

(N) UCSC genome browser view of an E2 responsive cMYC enhancer, Myc+135kb, showing colocalization with ESR1 binding site, H3K27ac and DHS site. The bidirectional signal of the enhancer is increased upon treatment with E2 and ATMi at 2h.

(O) Changes in *c-MYC* expression in response to E2 and ATMi at 2 h.

(P) eRNA expression at the c-MYC +135 kb enhancer in response to E2 and ATMi at 2 h.

Figure S5

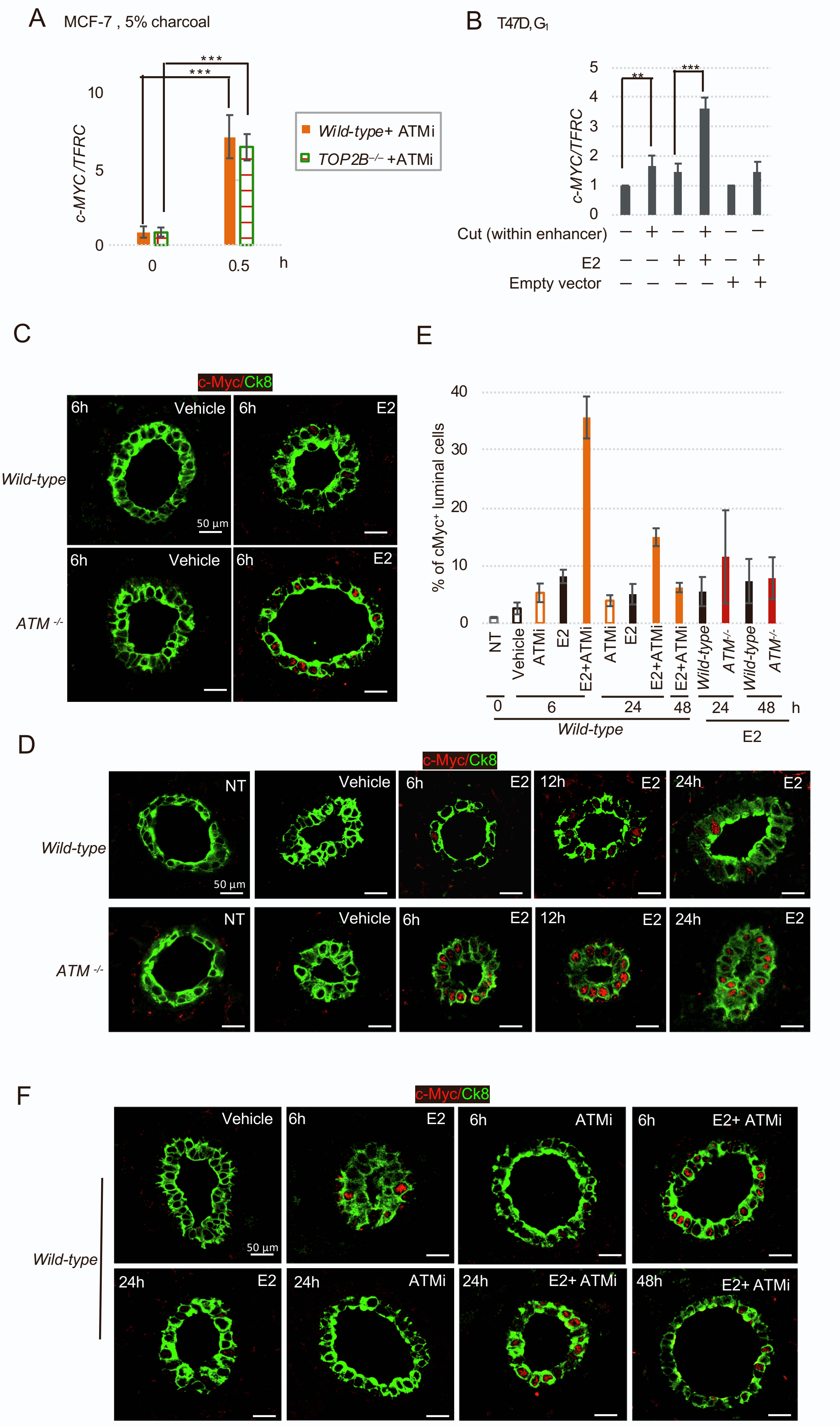

A
MCF-7 , 5% charcoal
c-MYC/TFRC
***
***
Wild-type+ ATMi
TOP2B-/- +ATMi
0
0.5
h
B
T47D, G1
c-MYC/TFRC
**
***
Cut (within enhancer)
E2
Empty vector
C
c-Myc/Ck8
6h
Vehicle
E2
Wild-type
ATM -/-
50 µm
E
% of cMyc+ luminal cells
NT
Vehicle
ATMi
E2
E2+ATMi
Wild-type
ATM-/-
0
6
24
48
h
Wild-type
E2
D
c-Myc/Ck8
NT
Vehicle
6h
12h
24h
E2
Wild-type
ATM -/-
50 µm
F
c-Myc/Ck8
Vehicle
6h
E2
ATMi
E2+ ATMi
24h
48h
Wild-type
50 µm

Figure S5. ATM loss increases the *c-MYC* transcriptional response to E2 in ER$^+$ human BC and murine mammary epithelial cells, Related to Figure 5

(A) *c-MYC* transcription in the indicated MCF-7 cells cultured with serum following addition of E2 at time zero. The amounts of *c-MYC* transcripts are normalized against the *TFRC* gene. Data represent the mean ± SD of three independent experiments. ***$P < 0.005$, Student's *t*-test.

(B) Quantification of the *c-MYC* mRNA level in T47D cells following cleavage at the *c-MYC* E-67 enhancer. The experiment was performed as in Figure 5 E.

(C) Cross-section of mammary ducts of female B6 mice carrying indicated genotypes showing representative images of c-Myc$^+$ epithelial cells of the mammary gland at 6 h after an intraperitoneal (i.p.) injection of E2 or solvent. Data are presented as in Figure 1A except the cells are immunostained for c-Myc rather than 53BP1.CK8 is a marker of epithelial cells. Quantification is shown in Figure 5 F. Scale bar represents 50 μm.

(D) A representative image for Figure 5G of B6;129 mice carrying the indicated genotypes, showing c-Myc$^+$ mammary epithelial cells at 6,12, 24, h after an intraperitoneal (i.p.) injection of E2 or solvent. Scale bar represents 50 μm.

(E) Percentage of c-Myc$^+$ cells after i.p. injection of E2 and/or ATMi into mice. Mammary glands were harvested 0, 6, 24, and 48 h after injection. The first nine datasets from *wild-type* B6 mice were replotted from Figure 5H. The last four datasets were obtained from $ATM^{-/-}$ and their age-matched *wild-type* controls from the same strain on the B6 background. Data represent mean ± SD from duplicates for $ATM^{-/-}$ and triplicates for the others.

(F) Representative image for Figure 5H showing c-Myc$^+$ mammary epithelial cells in the *wild-type* B6 mice after i.p. injection of E2 and ATMi. Scale bar represents 50 μm.

Figure S6

Three days experiments

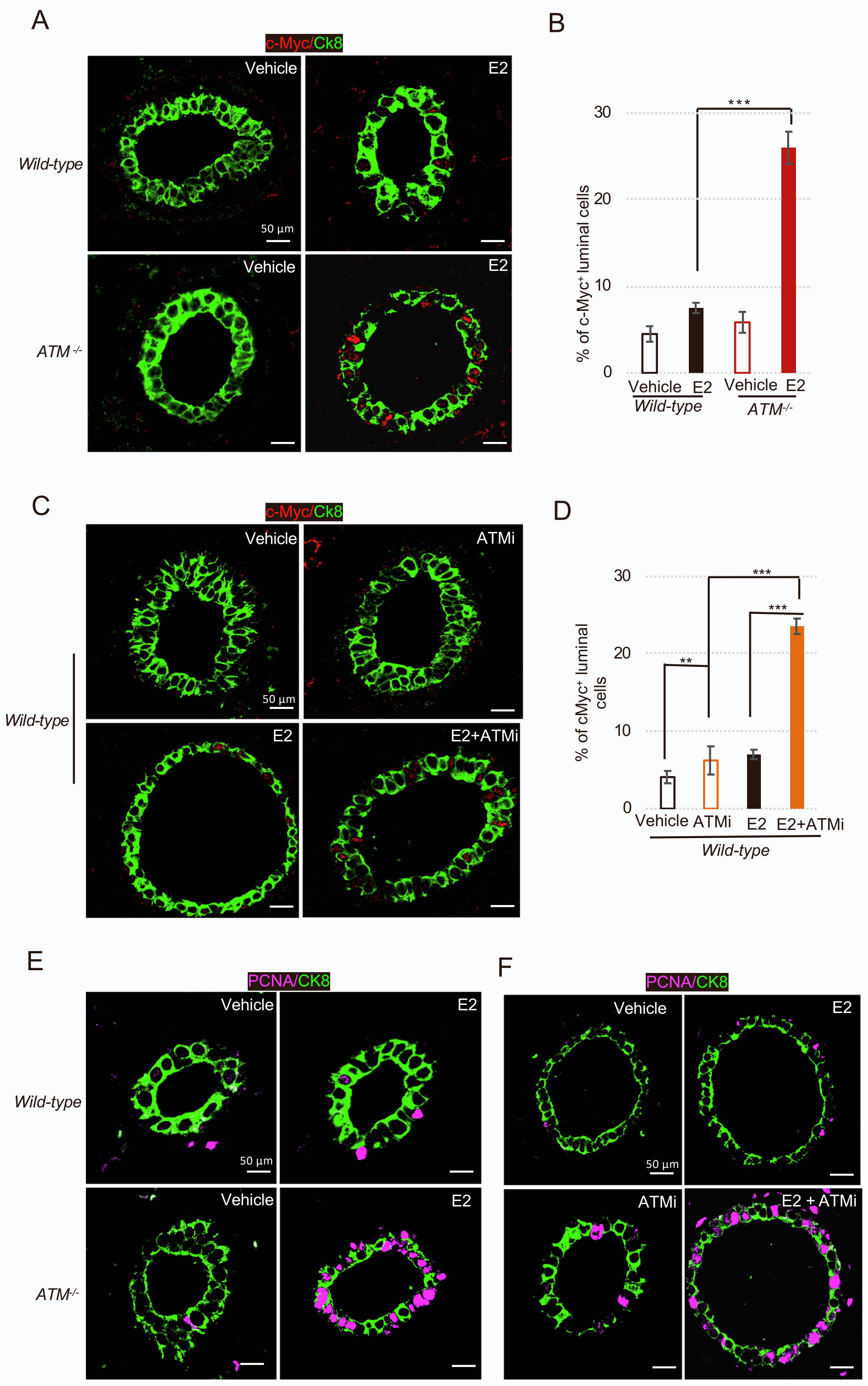

Figure S6. ATM loss causes abnormal proliferation following daily injection of E2, related to Figure 6

(A) Representative image showing c-Myc$^+$ mammary epithelial cells after daily intraperitoneal (i.p.) injection of E2 or solvent for 3 days into *wild-type* B6 mice having the indicated genotypes. The experiment was performed according to the scheme shown in Figure 6A. The quantification is shown in (B).

(B) Percentage of c-Myc$^+$ mammary cells in the indicated genotypes in (A) after daily intraperitoneal (i.p.) injection of E2 or solvent for 3 days. Quantification was performed as in Figure 5F, from triplicated experiments.

(C) Representative image of c-Myc$^+$ mammary epithelial cells in the *wild-type* B6 mice after daily intraperitoneal (i.p.) injection of E2 and ATMi for 3 days. Quantification is shown in (D).

(D) Percentage of c-Myc$^+$ mammary cells for the indicated genotypes in (C) after daily intraperitoneal (i.p.) injection of E2 or solvent for 3 days. Quantification was performed as in Figure 5F, from triplicated experiments.

(E) Representative images for Figure 6D, shows the percentage of PCNA$^+$ mammary epithelial cells after daily intraperitoneal (i.p.) injection in B6 mice of indicated genotypes with E2 or solvent for 3 days. Experiment was performed according to the scheme shown in Figure 6A.

(F) Representative images for Figure 6E, shows the percentage of PCNA$^+$ mammary epithelial cells after daily intraperitoneal (i.p.) injection of *wild-type* B6 mice with E2 or ATMi for 3 days.

(A, C, E, and F) Scale bar represents 50 μm.

SUPPLEMENTARY TABLES

Table S1. Oligonucleotide (continued from Key Resource Table)

REAGENT or RESOURCE	SOURCE	IDENTIFIER
TK6, *ATM* (gene disruption), Forward, left arm 5′-GCGAATTGGGTACCGGGCCgatatgtactttaggcctca-3′	This study	N/A
TK6, *ATM* (gene disruption), Reverse, left arm 5′-CTGGGCTGAGGGGGGCCGATTGTGAAAATAGCTTTGG-3′	This study	N/A
TK6, *ATM* (gene disruption), Forward, right arm 5′-TGGGAGCTTGTCGACTTAA gtacatttttcccagattt-3′	This study	N/A
TK6, *ATM* (gene disruption), Reverse, right arm 5′-CACAgtaggcgcgccttaaAAACAGCAAAGATACAAATT-3′	This study	N/A
TK6, *CtIP* $^{T847/859A}$, (Knock-In), Forward, left arm 5′-CTCACTATAGGGCGAATTGGGTACCGGGCCctaaggtcatctcatctcatc-3′	This study	N/A
TK6, *CtIP* $^{T847/859A}$, (Knock-In), Reverse, left arm, 5′-CTAGCGGGCCGCTGGGCTCGAGGGGGGCCTTGTATTTTCAAAATTGTAT-3′	This study	N/A
TK6, *CtIP* $^{T847/859A}$, (Knock-In) Forward, right arm, 5′-TGGGAAGCTTGTCGACTTAAtttcccacatattgtaaaca-3′	This study	N/A
TK6, *CtIP* $^{T847/859A}$, (Knock-In) Reverse, right arm, 5′-CACTAgtaggcgcgccttaaagctcacagggttgaggatc-3′	This study	N/A
TK6, *CtIP* $^{T847/859E}$, (overexpression), Forward primer 5′-TGCCGCCAGAACACAGGACCGGCCGTCCCACCATCGGGCGCG-3′	This study	N/A
TK6, *CtIP* $^{T847/859E}$, (overexpression), Reverse primer 5′-GAAGTTTTTGCGCCGGATCTtatgtcttctgctccttgcc-3′	This study	N/A
NEO^{R}_F (for genotyping), 5′-AACCTGCGTGCAATCCATCTTGTTCAATGG -3′	This study	N/A

PURO^R_F (for genotyping PCR), 5′-GTGAGGAAGAGTTCTTGCAGCTCGGTGA-3′	This study	N/A
TK6, *CtIP* $^{T847/859A}$, (Knock-In), Left arm sequencing primer_1 5′-CTCAAGTGTAAAGTGAAGGAATCGGCTGGA-3′	This study	N/A
TK6, *CtIP* $^{T847/859A}$, (Knock-In), Left arm sequencing primer_2 5′-TAGTAGGCTTTGATCCTAACTGAAATCTT-3′	This study	N/A
TK6, *CtIP* $^{T847/859A}$, (Knock-In), Left arm sequencing primer_3 5′-TATTATGCAGTATGCCAGC-3′	This study	N/A
CtIP $^{T847/859E}$ _F (for genotyping PCR) 5′-ATGAACATCTTGGGAAGCAG-3′	This study	N/A
TK6, *CtIP* $^{T847/859A}$, (Knock-In) Right arm sequencing primer_1: 5′-CTTCCCTTCCCAAGTTGTTTATTC-3′	This study	N/A
TK6, *CtIP* $^{T847/859A}$, (Knock-In) Right arm sequencing primer_2: 5′- GCGGAGGTTGCAGTGAGTGAG-3′	This study	N/A
TK6, *CtIP* $^{T847/859A}$, (Knock-In) Right arm sequencing primer_3: 5′- GCGGAGGTTGCAGTGAGTGAG-3′	This study	N/A
cMYC (qPCR)_Forward (for mRNA quantification) 5′-CCTGGTGCTCCATGAGGAGAC-3′	This study	N/A
cMYC (qPCR)_Reverse (for mRNA quantification) 5′-CAGACTCTGACCTTTTGCCAGG-3′	This study	N/A
TFRC (qPCR)_Forward (for mRNA quantification) 5′-ATCGGTTGGTGCCACTGAATGG-3′	This study	N/A
TFRC (qPCR)_ Forward (for mRNA quantification) 5′-ACAACAGTGGGCTGGCAGAAAC-3′	This study	N/A
BglII (1st primer pair) for 3C analysis Forward-5′-GAGGCAGCAACAGTGATTTTATTAG-3′ Reverse -5′-ACTTTTTGTAGAGAGGCAGTCTGG-3′	This study	N/A
BglII (2nd primer pair) for 3C analysis Forward-5′-TTATTAGGGGGCAGAGGGTGAGTG-3′	This study	N/A

Reverse -5′-TTAAAGCGATCCTTCGGCCTTTGC-3′		
BglII (sequencing primer) for checking interaction of 3C<br>5′-TTCATCCGGAGATGCTCC-3′	This study	N/A
E-67 gH2AX ChiP<br>Forward-5′-ACCACTCCCTAAACTTGCTC-3′<br>Reverse -5′-TCTTGTCCTGTCTACTCTGTC-3′	This study	N/A
gRNAs for Myc +135 kb enhancer cut<br>Forward-5′-GGGGATTGAGGGAAGTGGAG-3′<br>Reverse -5′- CTCCACTTCCCTCAATCCCC-3′	This study	N/A
Negative control_1_gRNAs_Myc +135 Enhancer cut<br>Forward-5′-CACCGTTACAACAAATCGGACTAT -3′<br>Reverse -5′- AAACATAGTCCGATTTGTTGTAAC-3′	This study	N/A
Negative control_2_gRNAs_Myc +135 Enhancer cut<br>Forward-5′-CACCGCTCCCAGTTAGGCTATTCCG -3′<br>Reverse -5′- AAACCGGAATAGCCTAACTGGGAGC-3′	This study	N/A
gRNAs for Myc E-67 enhancer cut<br>Forward-5′-CACCGTTTCCCAGATGCAGAGAACG -3′<br>Reverse -5′- AAACCGTTCTCTGCATCTGGGAAAC-3′	This study	N/A

Table S2. NET-CAGE mapping statistics

**Genotype, treatment, replicate**	**No. of reads**	**No. of reads mapped uniquely**
wild type, 0 h, replicate 1	19,479,538	14,910,511
wild type, 1 h, replicate 1	20,287,795	15,628,528
TDP2$^{-/-}$, 0 h, replicate 1	20,322,063	15,077,294
TDP2$^{-/-}$, 1 h, replicate 1	20,262,163	15,465,470
TDP2$^{-/-}$ + Mock, 0 h, replicate 1	20,299,347	16,109,595
TDP2$^{-/-}$ + Mock, 0 h, replicate 2	20,305,678	15,584,780
TDP2$^{-/-}$ + Mock, 0 h, replicate 3	20,246,828	16,187,779
TDP2$^{-/-}$ + Mock, 0.5 h, replicate 1	19,702,724	15,918,272
TDP2$^{-/-}$ + Mock, 0.5 h, replicate 2	20,558,431	16,307,424

TDP2$^{-/-}$ + Mock, 0.5 h, replicate 3	20,553,286	16,326,191
TDP2$^{-/-}$ + Mock, 1 h, replicate 1	21,308,018	16,501,712
TDP2$^{-/-}$ + Mock, 1 h, replicate 2	20,207,552	16,094,864
TDP2$^{-/-}$ + Mock, 1 h, replicate 3	20,392,383	16,206,888
TDP2$^{-/-}$ + Mock, 3 h, replicate 1	21,610,098	17,413,741
TDP2$^{-/-}$ + Mock, 3 h, replicate 2	20,056,298	16,646,243
TDP2$^{-/-}$ + Mock, 3 h, replicate 3	21,951,392	17,411,718
TDP2$^{-/-}$ + Mock, 6 h, replicate 1	20,893,860	16,549,911
TDP2$^{-/-}$ + Mock, 6 h, replicate 2	21,255,743	15,367,233
TDP2$^{-/-}$ + Mock, 6 h, replicate 3	21,111,422	15,589,809
TDP2$^{-/-}$ + TDP2, 0 h, replicate 1	21,631,306	17,678,936
TDP2$^{-/-}$ + TDP2, 0 h, replicate 2	21,604,115	17,487,300
TDP2$^{-/-}$ + TDP2, 0 h, replicate 3	21,862,476	18,265,795
TDP2$^{-/-}$ + TDP2, 0.5 h, replicate 1	21,633,197	17,797,634
TDP2$^{-/-}$ + TDP2, 0.5 h, replicate 2	21,653,711	17,302,229
TDP2$^{-/-}$ + TDP2, 0.5 h, replicate 3	21,175,150	17,053,249
TDP2$^{-/-}$ + TDP2, 1 h, replicate 1	20,455,478	16,451,650
TDP2$^{-/-}$ + TDP2, 1 h, replicate 2	19,721,316	15,597,621
TDP2$^{-/-}$ + TDP2, 1 h, replicate 3	21,002,431	16,573,864
TDP2$^{-/-}$ + TDP2, 3 h, replicate 1	20,262,965	15,464,559
TDP2$^{-/-}$ + TDP2, 3 h, replicate 2	19,959,554	15,960,133
TDP2$^{-/-}$ + TDP2, 3 h, replicate 3	20,573,566	16,946,648
TDP2$^{-/-}$ + TDP2, 6 h, replicate 1	21,350,791	17,336,199
TDP2$^{-/-}$ + TDP2, 6 h, replicate 2	20,707,498	16,845,835
TDP2$^{-/-}$ + TDP2, 6 h, replicate 3	21,675,646	17,581,643
TDP2$^{-/-}$ + dTDP2, 0 h, replicate 1	21,918,300	17,523,172
TDP2$^{-/-}$ + dTDP2, 1 h, replicate 1	21,160,130	16,759,779
wild type + DMSO + ethanol, 2 h, rep 1	20,150,550	15,868,549
wild type + DMSO + E2, 2 h, rep 1	19,862,045	15,803,542
wild type + ATMi + ethanol, 2 h, rep 1	19,219,951	14,856,240

wild type + ATMi + E2, 2 h, rep 1	19,392,654	15,665,934

SUPPLEMENTAL REFERENCES

S1. Sasanuma, H., Tsuda, M., Morimoto, S., Saha, L.K., Rahman, M.M., Kiyooka, Y., Fujiike, H., Cherniack, A.D., Itou, J., Moreu, E.C., et al. (2018). BRCA1 ensures genome integrity by eliminating estrogen-induced pathological topoisomerase II-DNA complexes. Proceedings of the National Academy of Sciences of the United States of America *115*, E10642-E10651. 10.1073/pnas.1803177115.
